# Effects of a Powered Knee–Ankle Prosthesis on Intact Joint Biomechanics Across Sustained Activities of Daily Life: A Case Series

**DOI:** 10.1109/TNSRE.2026.3659043

**Published:** 2026

**Authors:** Emily G. Keller, Curt A. Laubscher, Robert D. Gregg

**Affiliations:** The authors are with the Department of Robotics, University of Michigan, Ann Arbor, MI 48109 USA.

**Keywords:** Prosthetics, biomechanics, gait, rehabilitation robotics, wearable robots

## Abstract

Lower-limb prosthesis users often overuse their intact joints due to the lack of positive work generated by their devices. This overreliance has been shown to increase joint loading, degeneration, and pain. While powered prostheses can generate positive work and therefore reduce this burden, clinical studies of commercialized single-joint devices have demonstrated inconsistent results. Recently, prototype powered knee *and* ankle prostheses have shown more consistent advantages over passive devices in laboratory settings. Most of the studies, however, focus on the biomechanics of the prosthesis rather than its impact on the user’s joints, study isolated activities, and/or do not replicate the demands of continuous real-world use. This case series analyzes the intact joint moments and work for N=3 above-knee amputee subjects using a powered knee-ankle prosthesis vs. their prescribed passive device during a continuous, sustained sequence of the primary activities of daily life. The powered prosthesis decreased peak hip flexion moment (but increased peak extension moment) during level walking, and decreased peak knee extension moment for all other activities. For at least two of the three subjects, the powered prosthesis decreased total positive work across the intact joints during ascent activities (stair ascent, sit-to-stand) and decreased negative total work for descent activities (stair descent, stand-to-sit). This case series suggests that powered knee-ankle prostheses have the potential to reduce overuse of intact joints in emulated real-world conditions.

## I. Introduction

PEOPLE with lower-limb amputations rely on their intact joints to compensate for lost function when using conventional prosthetic devices, causing an increase in joint loading [1], [2], [3], [4], [5], [6]. This overuse of intact joints often leads to joint degeneration [7], [8] and pain [7], [9] — outcomes that could significantly impact mobility and quality of life. While conventional prostheses can only do net negative work, powered prostheses can perform net positive work to potentially lessen the burden on the intact joints.

Single-joint powered prostheses, including the Power Knee^™^ (Össur, Reykjavík, Iceland) and Empower^™^ ankle (Ottobock, Duderstadt, Germany), have entered clinical use since 2006. When compared to passive devices, these commercial powered devices have enabled increased walking speed [10], [11] and improved symmetry in peak ground reaction forces [5], [12] and hip moments [13] during sitting and standing. However, other results are mixed, showing improvements alongside null [13] or even detrimental results [6], [14] in terms of intact joint kinetics. In one study, all subjects preferred a microprocessor-controlled knee over their usual mechanical knee, whereas four out of ten subjects requested to stop using the powered knee [15]. These subjects cited weight, noise, and challenges with operation as reasons for discontinuing use, which may partly explain the poor adoption rates of powered devices.

Despite these challenges with commercial (single-joint) powered prostheses, research on prototype powered *knee-ankle* prostheses has demonstrated many potential benefits. These dual-joint prostheses have reduced metabolic cost during stair ascent [16], improved ground reaction force symmetry during sitting and standing [17], [18], reduced hip compensations [19] and back muscle activation [20] during walking, and demonstrated some reductions in biological joint work during ramp and stair navigation [21]. However, the benefits of powered knee-ankle prostheses have not yet justified the additional complexity, cost, and weight of these devices, for which commercial translation has remained elusive. Most studies on powered knee-ankle prostheses focus on one or more isolated activities performed over a short duration [16], [19], [20], [21], [22]. While important, these studies fall short of capturing the potential benefits of powered prostheses in real-world use cases, which require continuous activity sequences with automatic detection of transitions over an extended period of time. A recent study [23] considered these factors but focused on the behavior of the prosthetic device itself rather than the intact joints. To fully justify the clinical use of powered knee-ankle prostheses, a better understanding is needed of their impact on users’ intact joints during real-world use cases.

While comprehensive real-world testing of powered knee-ankle prostheses was previously limited by insufficiently adaptable or reliable control methods, recent advances are now making this research possible. Adaptable mid-level controllers have been introduced to enable continuous variations of activities (e.g., variable speed/incline walking) and reduce the number of distinct activity modes requiring classification [18], [24], [25], [26], [27], [28]. To bypass the classification problem, researchers manually initiated activity transitions between four adaptable mid-level controllers in an N=1 endurance case study [29], showing that the powered knee-ankle prosthesis enabled the subject to complete more laps of a multi-activity circuit than with their conventional prosthesis. To enable more practical testing scenarios, Cheng et al. subsequently introduced an automatic classification system for the four adaptable activity modes, achieving >99% classification accuracy and 100% recovery from misclassifications while navigating a multi-activity circuit over long durations [23]. With this controller, studies can now be more reflective of real-world community ambulation to demonstrate the possible benefits of powered knee-ankle prostheses.

**Fig. 1.**
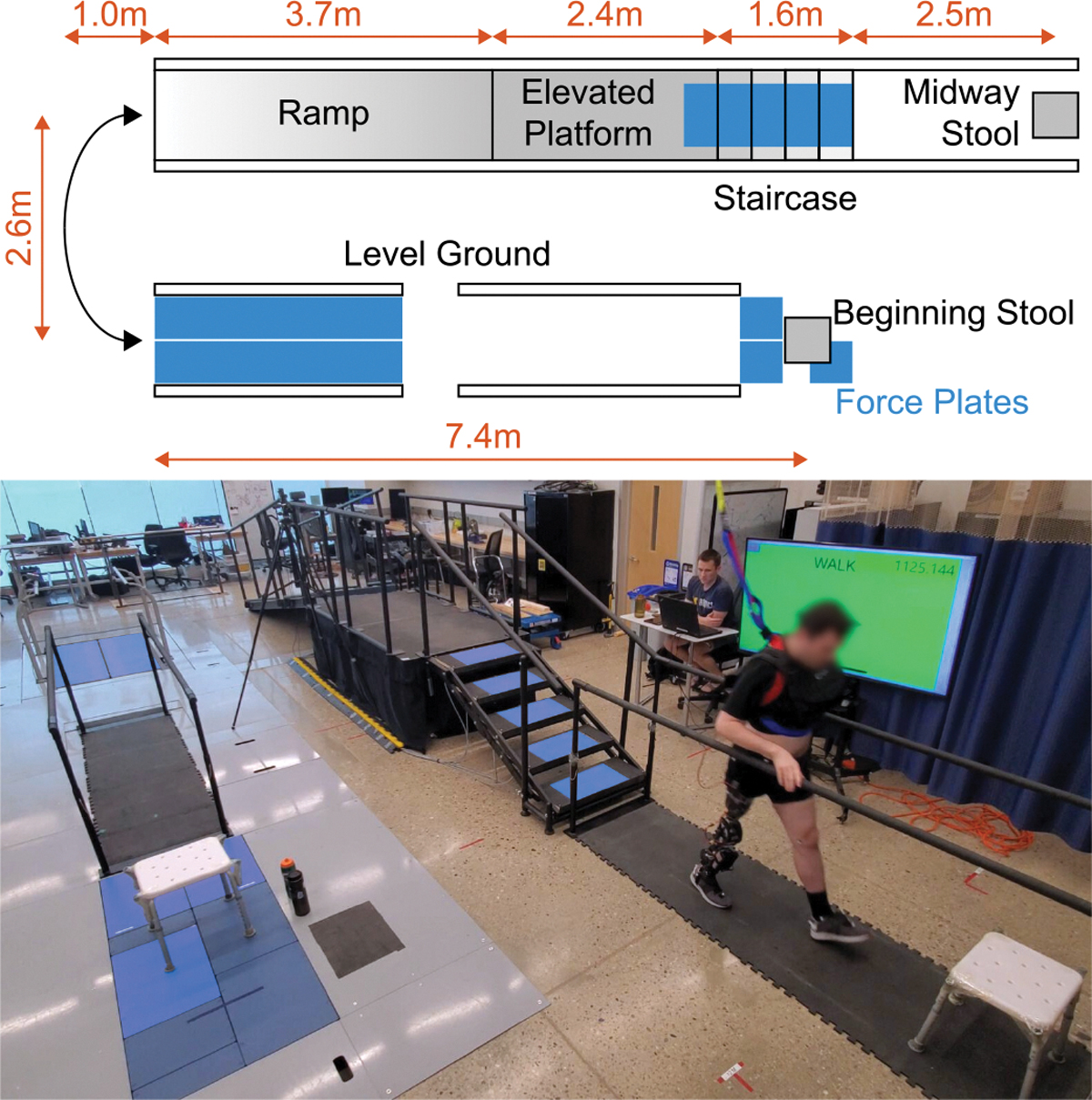
Diagram (top) and photo (bottom) of the multi-activity circuit in this study. Force plates were located underneath and in front of the beginning stool, on each stair, and between the parallel bars on level ground (highlighted in blue). A handrail was also placed on the left edge of the diagram (far end of the bottom image) for safety while turning.

In this study, we present a comprehensive exploratory analysis of the kinetics and energetics of the intact lower-limb joints during a sustained, multi-activity experiment, comparing use of a powered knee-ankle prosthesis with each subject’s passive daily-use microprocessor prosthesis. Three high-mobility (K4) above-knee amputee subjects continuously and repeatedly traversed a circuit (Fig. 1) with automatic transitions between the primary activities of daily living to better represent real-world ambulation. While the improvements were not universal, these three subjects experienced some meaningful benefits to their intact limb kinetics and energetics with the powered prosthesis. For most or all subjects, the powered prosthesis decreased the peak flexion moment in the device-side hip during walking and the peak extension moment in the intact-side knee during sit-to-stand, stand-to-sit, stair ascent, and stair descent. The powered device also led to marked reductions in total positive work of the intact joints during stair ascent and sit-to-stand, as well as reduced total negative work during stair descent and stand-to-sit. This suggests that the powered knee-ankle prosthesis can lessen the loads on intact joints compared to passive prostheses, which may prevent secondary degenerative disorders for improved quality of life.

## II. Methods

The study protocol (HUM00230065) was approved by the Institutional Review Board at the University of Michigan. This study involved multiple visits to a gait laboratory, including at least six acclimation sessions and two data collection sessions (one with the passive device and one with the powered device). While five above-knee amputee subjects provided written consent to participate, only three completed the multi-day study protocol — one participant dropped out due to residual limb pain and the other due to personal reasons. Incomplete data were dropped from the analysis. Each subject wore their daily-use device for the passive prosthesis condition of the experiment. Details for each subject and their prosthetic devices are provided in Table I. A practicing, certified, licensed prosthetist was present at the first acclimation session with the powered knee-ankle prosthesis to ensure proper fitting and alignment. The following sections describe the powered device, experimental methods, and data collection/analysis.

### A. Powered Prosthesis Hardware and Control.

**Fig. 2.**
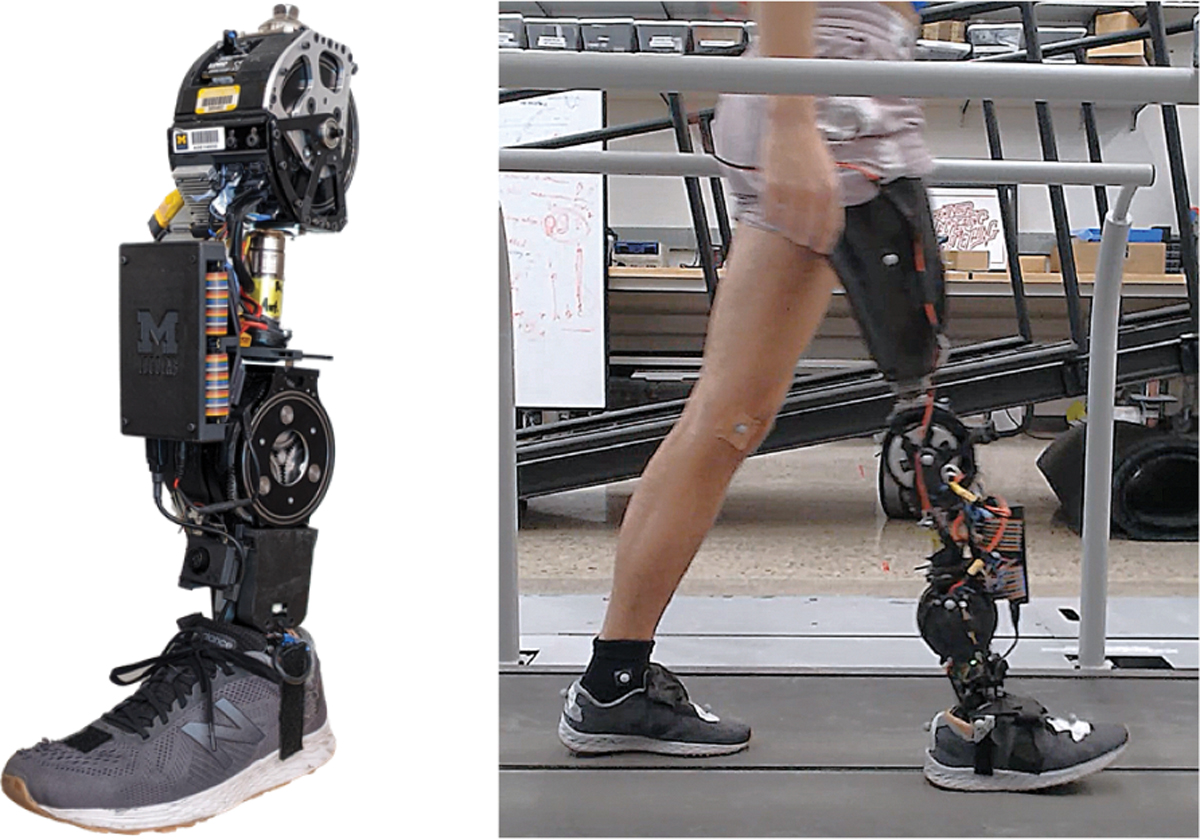
Left: image of the powered knee-ankle prosthesis used in this study. Right: a subject wearing the device and motion capture markers while walking over the stationary Bertec treadmill (used as force plates).

The powered knee-ankle prosthesis used in this study is shown in Fig. 2 with detailed specifications given in [30]. The weight of the device is 5.66 kg, not including the batteries worn on the subject’s back in a harness. Each joint of the device can provide a peak torque of 180 Nm via low-impedance actuators, which also allow accurate open-loop torque control and backdrivability (e.g., ballistic swing motion) [30]. Joint angles are measured by motor encoders, and thigh and foot angles are measured by respective IMUs (3DM-CX5–25, HBK MicroStrain, Williston, VT, USA). An ultrasonic distance sensor (LV-MaxSonar-EZ4, MaxBotix, Brainerd, MN) attached above the ankle enables automatic activity classification [23] as well as stub avoidance [31].

The control architecture used on the powered device was developed based on reference kinematics and kinetics from unimpaired population data, as detailed in [23]. The high-level activity classifier uses normative kinematic cues and distance perception to automatically select between mid-level controllers for four activity modes: sit/stand [18], walk [26], stair ascent [27], and stair descent [27]. The walk controller continuously adapts to ramps using real-time slope detection [26], eliminating the need for distinct ramp ascent/descent controllers. The high-level and mid-level controllers facilitate activity transitions led by either the intact or prosthetic side.

Each of the activity modes has a similar structure: a phase-based hybrid kinematic-impedance controller that tracks an angular position trajectory during swing phase and renders variable impedance during stance phase. For the impedance controller, torque τ is calculated as a function of stiffness K, damping B, equilibrium angle $\theta_{\text{eq}}$, and joint angle θ as shown:

$$\tau =K\left(s,\chi ,\mu \right)\left[\theta_{eq}\left(s,\chi ,\mu \right)-\theta \right]-B\left(s,\chi ,\mu \right)\dot{\theta }.$$

Stiffness, damping, and equilibrium angle are functions of gait phase variable s, task χ (walking speed/incline or stair height), and activity mode μ (sit/stand, walk, stair ascent, or stair descent). In this study, the speed input to the walk controller was fixed at 1 m/s (as subjects were instructed to walk at a fixed speed), and the stair height of the stair controller was fixed at 5 in (12.7 cm).

The swing phase uses a position-derivative tracking controller, where gains $k_{p}$ and $k_{d}$ scale the position and velocity tracking error from a desired trajectory $\theta_{d}$, with a constant viscous damping coefficient β for stability:

$$\tau =k_{p}\left[\theta_{d}(s,\chi ,\mu )+\theta_{s}(z)-\theta \right]+k_{d}[{\dot{\theta }}_{d}(s,\chi ,\mu )-\dot{\theta }]-\beta \dot{\theta }.$$

The additional term $\theta_{s}(z)$ is added to adjust reference kinematics during stair traversal to avoid potential toe stubs detected by the ultrasonic distance z [31].

### B. Protocol.

The subjects traversed the circuit shown in Fig. 1, matching the setup in [29]. Subjects began seated, then performed sit-to-stand, level walking, incline walking (11.1 deg), stair descent (5 in height), and stand-to-sit with a midway stool. Subjects were instructed to complete the stand-to-sit movement fully, and then immediately initiate the sit-to-stand *without pausing*. To complete a lap, the subject then traversed the same track in the reverse direction, performing the sit-to-stand, then stair ascent, decline walking, level walking, and finally stand-to-sit, ending in the beginning stool again.

Each subject was acclimated to the powered device across multiple visits until they felt comfortable performing each activity and could reliably complete laps on the circuit. Then they were timed completing laps at a “brisk” pace, while not being allowed to run or skip stairs, with their passive and the powered device. From these two lap times — one for powered and one for passive — the slower of the two was multiplied by a 1.1 factor, and that time was used as their baseline. This 10% adjustment accounts for normal variability in lap completion times from occasional missteps or misclassifications, for example. On each data collection day (powered or passive), participants completed a few practice laps with the designated device prior to the main experiment to familiarize themselves with the target pace. Data collection involved continuously repeated laps, where participants were given feedback if they were too fast or too slow per lap. Trials lasted until subjects could no longer sustain their baseline time, up to a time limit for each subject. Each session lasted for at least 45 minutes (at least 48 laps), except S2 stopped after about 20 minutes (18 laps) in the powered condition due to fatigue and socket suction issues caused by device weight. The order of passive or powered condition was alternated between subjects.

### C. Data Collection and Processing.

During data collection, subjects were outfitted with motion capture markers and recorded using 26 Vicon infrared cameras (Vicon, Oxford, UK) at 250 Hz. Force plates throughout the lab allowed capture of ground reaction forces during level-ground walking, traversing the stairs, and performing sit-to-stand and stand-to-sit at the beginning stool. No force plates were located on the ramp or at the midway stool. Synchronous data acquisition was managed by the Vicon motion capture system. Marker data and ground reaction force data were processed using a modified version of Plug-in Gait as described in the Supplemental Methods to get kinematics and kinetics. The Supplemental Methods also describe how the mass, center of mass, and inertia of the thigh, shank, and foot of the amputated side were altered to reflect properties of the powered and passive devices.

Outlier strides were removed if either the duration between consecutive heel strikes was greater than 2 sec or the Variance Accounted For (VAF) of the kinematic and kinetic trajectories fell below 50%. This outlier detection effectively removed the strides associated with activity misclassifications, which were infrequent as this study achieved the same >99% classification accuracy as [23]. Transition strides were also discarded (8 strides per lap).

### D. Data Analysis.

The kinetics for the intact-side and device-side hips were analyzed in both the sagittal and frontal planes, where the latter relates to common compensatory movements. The intact knee was also analyzed in both planes, where knee adduction moment is strongly associated with osteoarthritis progression [32]. The intact ankle was only analyzed in the sagittal plane. Because the differences between powered and passive prosthetic joint kinetics have been well-documented in prior studies [18], [23], [26], [27], these variables are only presented in the Supplemental Material.

Intact joint moments and work were selected as the primary outcomes to highlight user effort. Frontal-plane knee work was not analyzed due to the lack of an articulated joint in this plane, but additional frontal-plane knee biomechanics are provided in the Supplemental Material. The Supplemental Material also provides the peak power for the studied joints, which is mathematically related to work. To investigate total lower-body work, three metrics were calculated: total negative (absorbed) work, total positive (generated) work, and weighted absolute work (as a proxy for total user effort). The positive and negative portions of joint power were separately integrated over time to obtain individual positive and negative work values. Total positive work was computed by summing the positive work values across the six joint axes, and total negative work by summing the negative work values. Weighted absolute work is 4.8 times total positive work plus the absolute value of total negative work [33].

One notable exception to these metrics was made for stair descent. When using a passive prosthesis, users are trained to place the foot over the edge of the step so that it may roll over to compensate for the lack of ankle dorsiflexion. This stair descent strategy caused the ground reaction force to be split between the front rail and force plate of our instrumented stairs, reducing force plate measurements (especially during late stance). This only impacted the device-side hip kinetics in the passive condition, but this measure was discarded from the primary analysis for both prosthesis conditions as they cannot be rigorously compared. Device-side hip kinetics are still reported in the Table S-IV in the Supplemental Material.

The stair ascent analysis was also impacted by different gait strategies between conditions, though this did not cause measurement artifacts. Two of the three subjects — S1 and S3 — used a step-over-step gait, where each step is only contacted by one foot, for both conditions. Although S2 also used a step-over-step gait in the powered condition (as required), they used a step-by-step approach with their passive device, lifting the intact leg to the next step and then bringing the device leg up to stand on the same step. Due to the difference in strategy, S2 was excluded from the primary analysis for this task, though values are still reported in Table S-V in the Supplemental Material.

To evaluate the *within-subject* effects of the powered device on biomechanical outcomes, a linear model with subject-specific fixed effects was used. Subjects S1, S2, and S3 were considered independently due to the small sample size, the exploratory nature of this case series, and the heterogeneity of population responses to different devices and activities [21]. The significance level was set at 0.05, and significant results that are relevant to a given activity are reported. Additional information on the model is given in Supplemental Methods. Note the total lower-body work metrics were not analyzed statistically but instead are reported if the difference between conditions exceeded 0.05 J/kg. While relevant or significant results are highlighted in the following section, complete results are included in the Supplemental Materials.

## III. Results

Unless otherwise stated, all reported changes in human joint biomechanics refer to significant differences in that subject’s powered condition relative to their passive condition. In the case of negative values, increases refer to the *magnitude*. The Supplemental Material contains the full catalog of results.

### A. Sit-To-Stand.

#### 1) Hips:

**Fig. 3.**
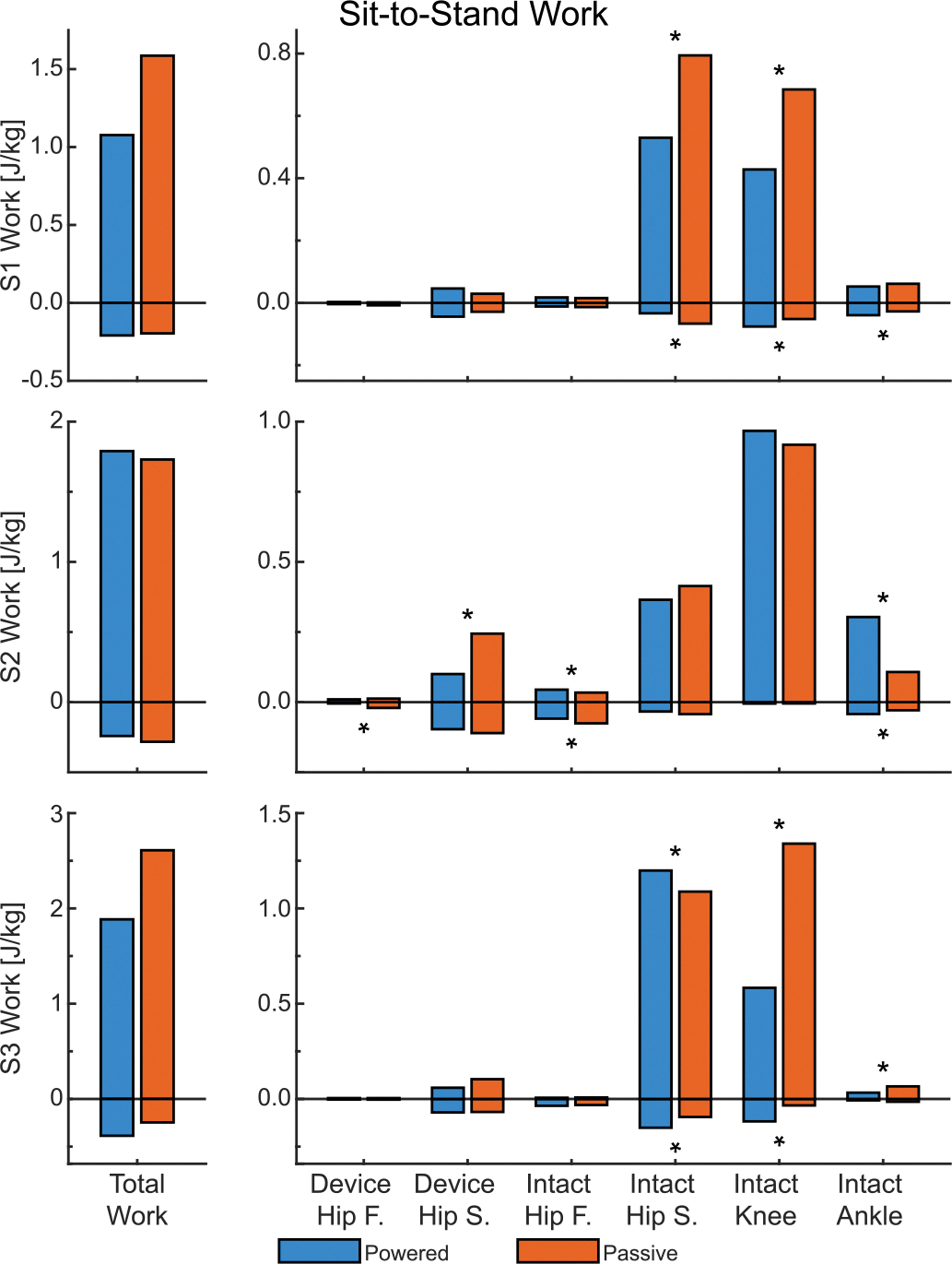
The total biological work and joint components for sit-to-stand. On the left is total work — the sum of the individual joint components on the right. Frontal plane is abbreviated as F and sagittal plane as S. An asterisks (*) denotes significant difference (*α* < 0.05) between the conditions. Significance was not tested for Total Work.

In the sagittal plane, hip extension moments (Fig. S6) and positive hip work (Fig. 3) were substantially lower on the device side than the intact side, regardless of condition. Comparisons between the powered and passive conditions were mixed (see Fig. S6 in Supplemental Material). The powered condition reduced the intact-side peak extension moment by 11.26% for S1, but S3 showed an increase of 25.76%. Similarly, intact-side positive work decreased by 32.28% for S1 — attributed to higher positive power in mid-to-late sit-to-stand — but increased by 10.17% for S3 (Fig. 3). On the device side, only S2 experienced a reduction in peak extension moment (by 23.30%), which contributed to a 58.99% reduction in positive work (along with decreased power beginning mid sit-to-stand, see Fig. S6 in Supplemental Material).

Hip flexion moments were smaller than extension moments, resulting in lower magnitudes of negative work than positive work (Fig. 3). Using the powered prosthesis, S1 decreased peak flexion moment on the device side and intact side by 62.10% and 55.94%, respectively, while joint velocities remained relatively consistent between prosthesis conditions. Therefore, the powered prosthesis facilitated a 49.75% decrease in intact-side negative work. Subject 3 decreased intact-side peak flexion moment by 68.80% but increased intact-side negative work by 59.93%.

Frontal-plane hip biomechanics play a minor role during sit-to-stand and as a result have very low kinetic magnitudes (Fig 3). Both the moments and works had mixed results with S1 exhibiting decreases in peak device-side moments and intact-side work, while S3 increased intact-side metrics.

#### 2) Intact Knee:

**Fig. 4.**
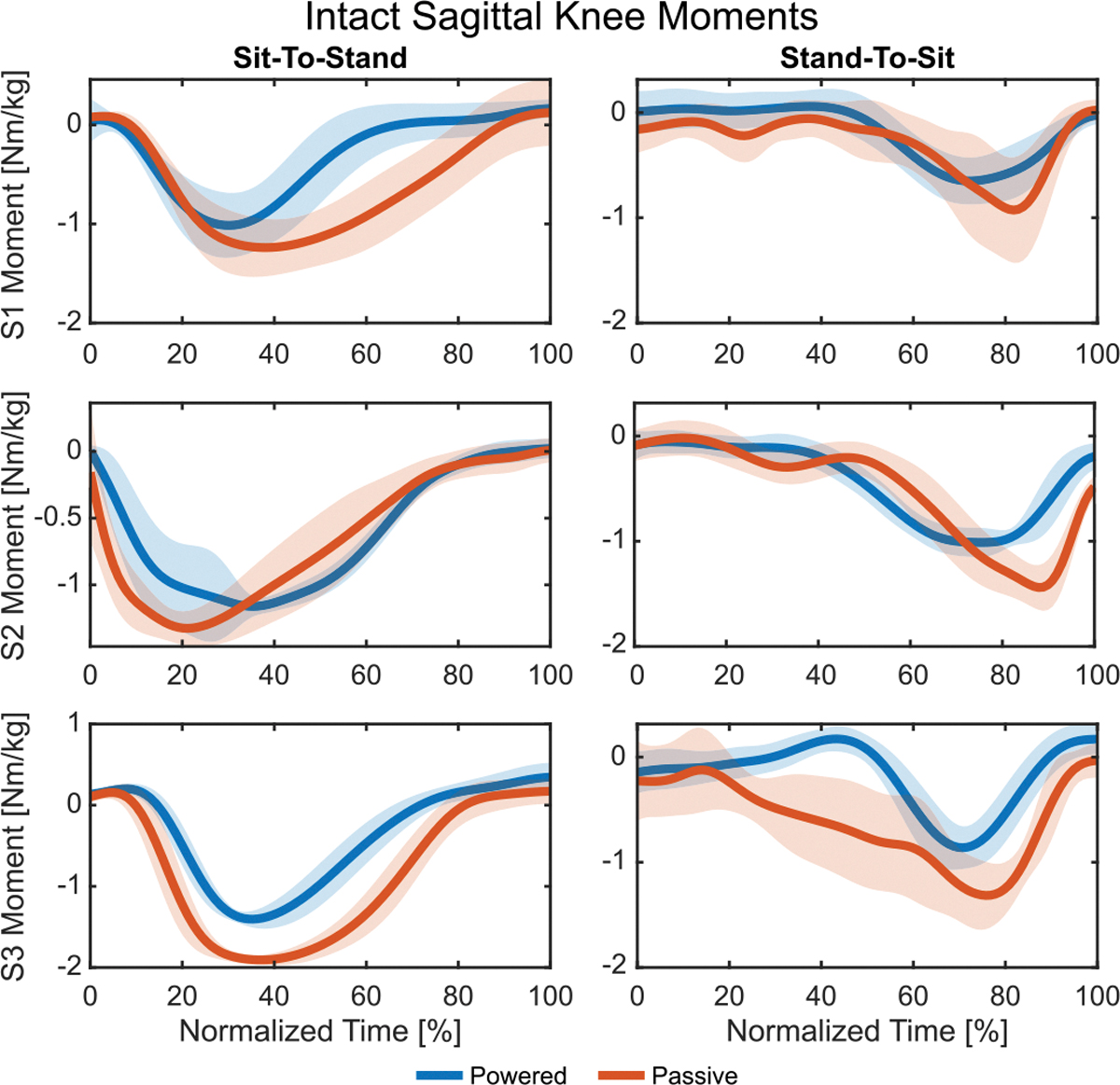
The average intact knee sagittal-plane moments for each subject during sit-to-stand (left) and stand-to-sit (right) motions. Flexion is positive and extension is negative. The powered condition is shown in blue and the passive condition in orange with shaded regions showing ±1 standard deviation. Intact knee moments are reduced in the powered case compared to passive for both activities.

The powered condition decreased the peak sagittal extension moment of the intact-side knee for all three subjects by an average of 19.21% (Fig. 4, left). As a result, positive sagittal-plane knee work decreased for S1 by 37.48% and S3 by 56.47% (Fig. 3). Peak knee abduction moments had a similar trend for S1 and S3 with reductions of 21.96% and 23.28%, respectively, while S3 increased by 60.61%.

#### 3) Intact Ankle:

Only S3 saw a significant (29.22%) decrease in peak plantarflexion moment, resulting in a 49.90% decrease in positive ankle work (Fig. 3). Because S2 sustained the plantarflexion moments for longer in the powered condition, they exhibited a 182.65% increase in positive ankle work, yielding a mixed result.

#### 4) Total Work:

The powered condition reduced total work metrics during sit-to-stand for two subjects (Fig. 3). S1 exhibited a 32.11% reduction in total positive work, contributing to a 31.14% reduction in weighted absolute work. Similarly, S3 had a 27.78% decrease in total positive work, leading to a 26.15% reduction in weighted absolute work (despite total negative work increasing by 56.60% with relatively small magnitude). In contrast, S2 increased total positive work by 3.49%, resulting in a 2.89% increase in weighted absolute work.

### B. Stand-To-Sit.

#### 1) Hips:

**Fig. 5.**
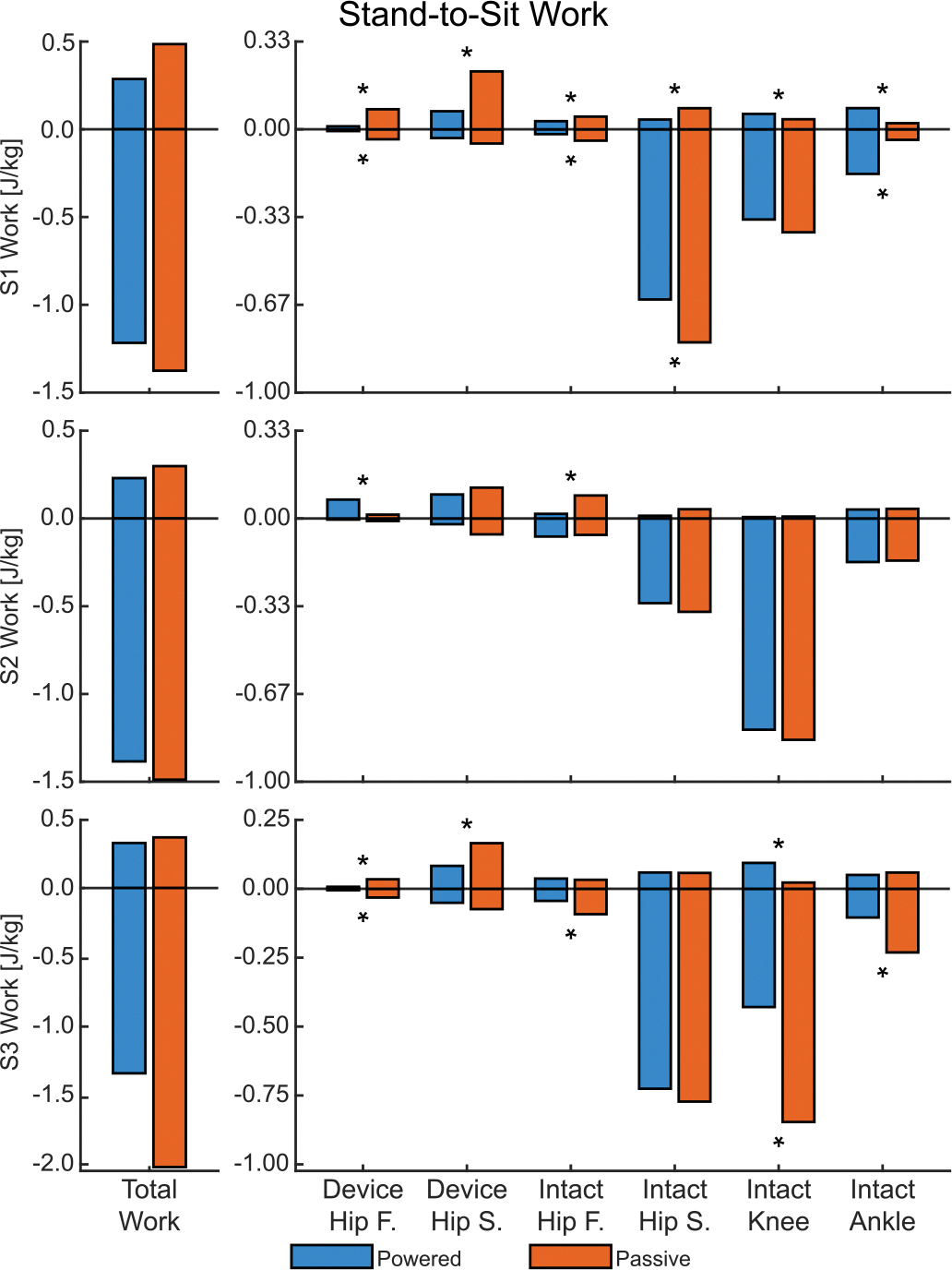
The total biological work and joint components for stand-to-sit. On the left is total work — the sum of the individual joint components on the right. Frontal plane is abbreviated as F and sagittal plane as S. An asterisks (*) denotes significant difference (*α* < 0.05) between the conditions. Significance was not tested for Total Work.

Peak hip extension moment showed mixed results. On the device side, S1 and S3 maintained low hip extension torques for both conditions. Subject 2 exhibited a late extension moment in the passive condition but not in the powered condition, resulting in a 91.39% reduction (see Fig. S7 in Supplemental Material). In contrast, both conditions for S2 and S3 exhibited an increasing trend in intact-side extension torque until late in the stand-to-sit transition, where S3 decreased peak values by 13.00% in the powered condition. Subject 1 was an exceptional case with low magnitudes in hip extension moment throughout the passive condition, causing a 121.12% increase for the powered condition. Negative hip work in the sagittal plane decreased on both sides for all subjects (Fig. 5), but only the 19.75% reduction on the intact side of S1 was significant.

Frontal plane hip biomechanics play a minor role during stand-to-sit and as a result have very low kinetic magnitudes (Fig 5). The powered condition decreased the peak adduction moment as well as both positive and negative work on the device side for S1 and S3, while S2 exhibited a larger peak adduction moment along with increased positive work.

#### 2) Intact Knee:

The powered condition decreased the intact-side knee’s peak sagittal-plane extension moment for all three subjects by an average of 28.47% (Fig. 4, right), and the peak consistently occurred earlier in late stand-to-sit than the passive condition. Negative sagittal-plane knee power saw a similar reduction and shift, leading to a reduction in negative sagittal-plane knee work for all subjects (Fig. 5), though only S3 was significant (49.29%). Frontal-plane peak abduction moments also decreased for all three subjects, while being significant only for S1 (15.71%) and S3 (33.53%).

#### 3) Intact Ankle:

The powered condition decreased the peak plantarflexion moment for S2 by 16.39% and S3 by 40.41%, but increased the moment for S1 by 112.99%. Negative ankle work in Fig. 5 had mixed results: S1 increased by 323.48% (attributed to the low baseline value for passive), whereas S3 decreased by 54.85%.

#### 4) Total Work:

All three subjects experienced reductions in total work metrics in the powered condition (Fig. 5). Total negative work decreased by an average of 17.31% for all three subjects, leading to an average 22.61% reduction in weighted absolute work due to the absorptive nature of stand-to-sit. Even so, total positive work still decreased for S1 by 40.96% and S2 by 22.85%.

### C. Level Walking.

#### 1) Hips:

**Fig. 6.**
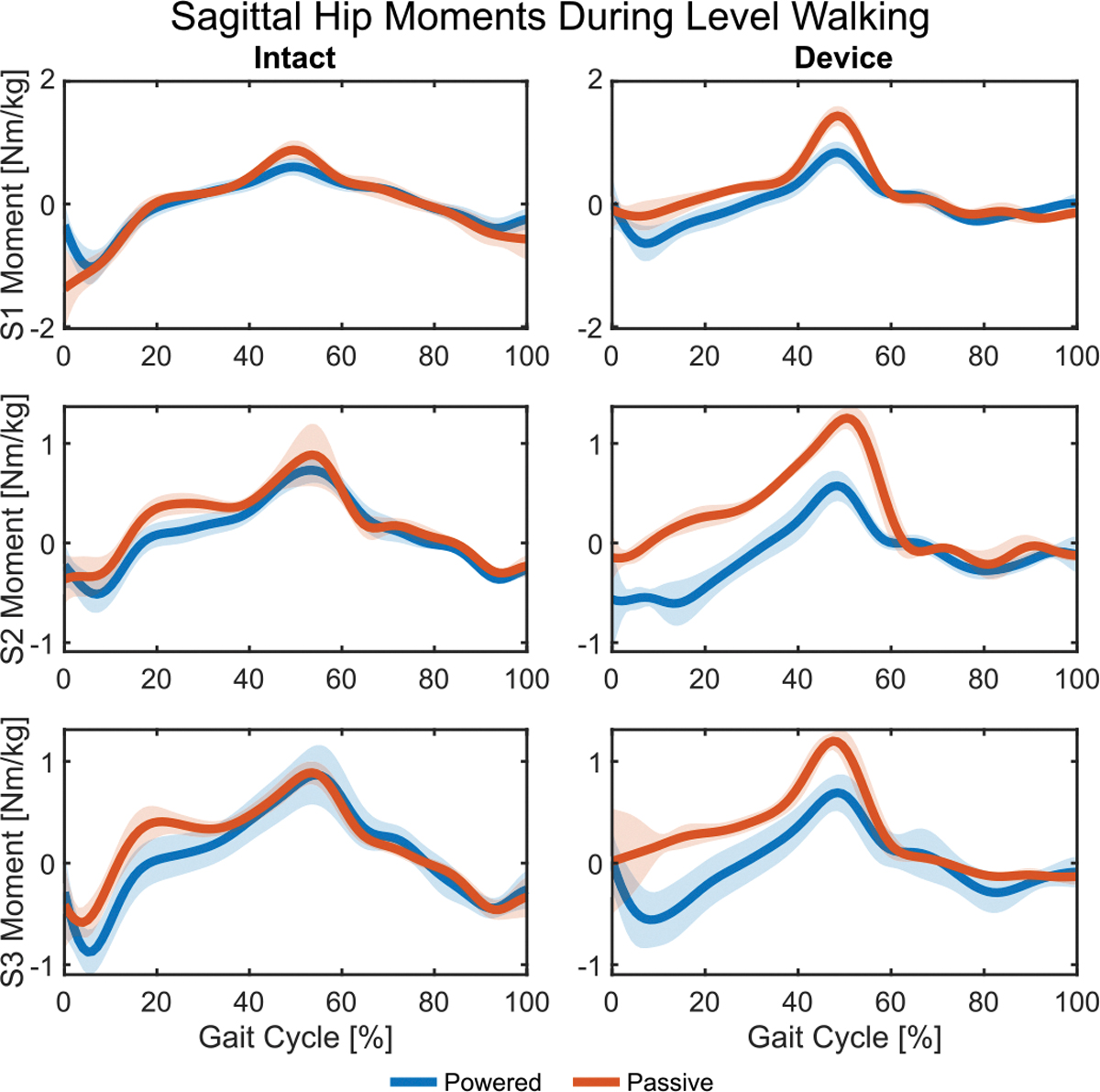
The average sagittal-plane hip moments during level walking for the intact (left) and device (right) side of each subject. Flexion is positive and extension is negative. The powered condition is shown in blue and the passive condition in orange with shaded regions showing ±1 standard deviation. Device-side hip flexion moments are greatly reduced in the powered case compared to passive.

**Fig. 7.**
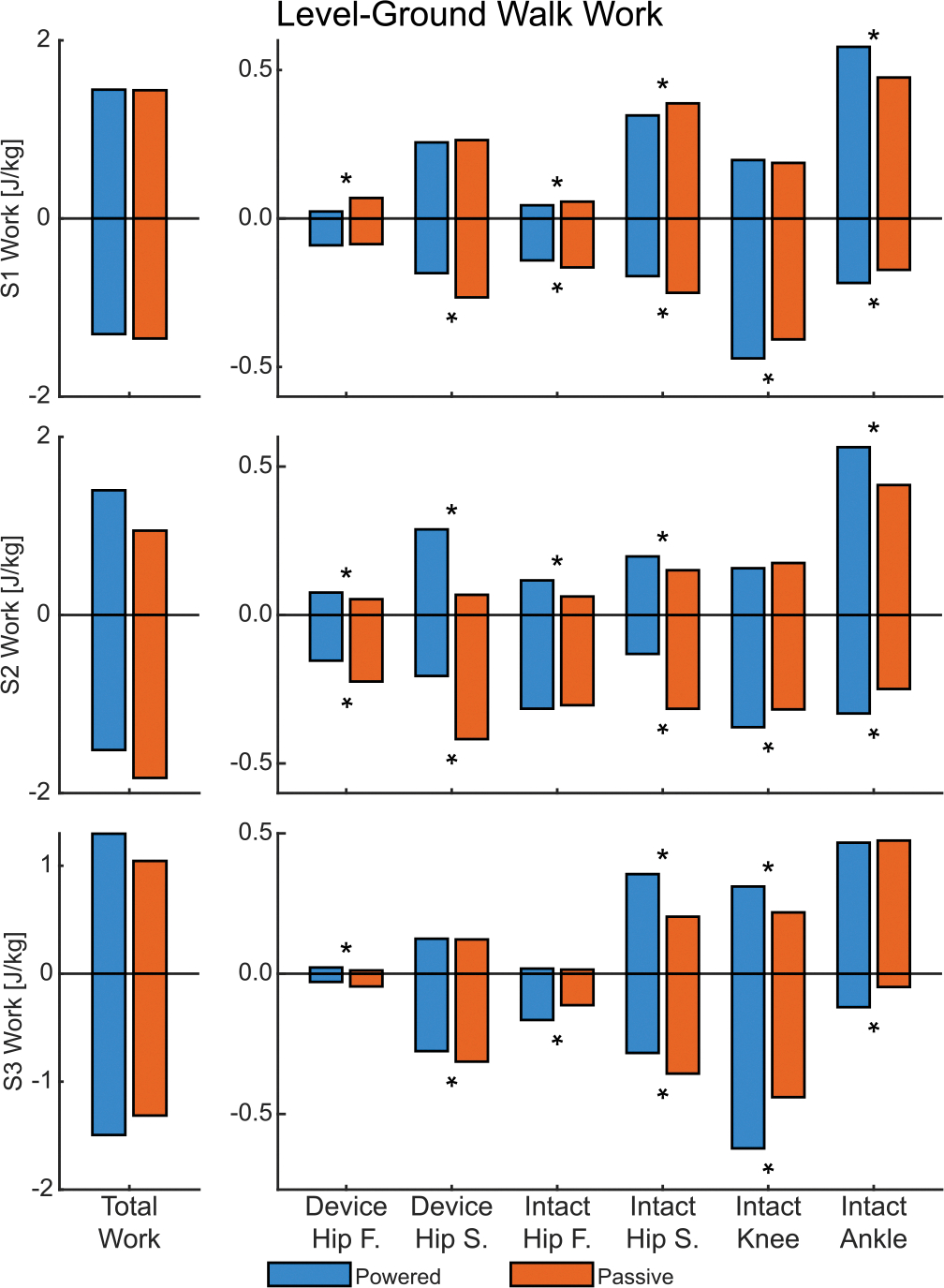
The total biological work and joint components for level-ground walking. On the left is total work — the sum of the individual joint components on the right. Frontal plane is abbreviated as F and sagittal plane as S. An asterisks (*) denotes significant difference (*α* < 0.05) between the conditions. Significance was not tested for Total Work.

The powered condition generally reduced peak hip flexion moments — responsible for pulling the leg into swing phase — and negative hip work in the sagittal plane — responsible for redirecting the body’s center of mass. The peak flexion moment decreased on the device side for all subjects by an average of 46.73% (Fig. 6, right) and on the intact side for S1 by 27.71% and S2 by 12.58% (Fig. 6, left). Given that joint velocities were similar between prosthesis conditions, these smaller joint moments led to smaller magnitudes of negative hip work in the sagittal plane for all three subjects, reduced by an average of 31.18% on the device side and 33.87% on the intact side (Fig. 7). Positive hip work, however, generally increased. On the device side, positive hip work increased for S2 by 324.19%, primarily due to low positive power in the passive condition. On the intact side, positive work increased for S2 by 30.85% and S3 by 74.54%, though decreased for S1 by 10.50%. Following that trend, peak hip extension moment on the intact side decreased for S1 by 32.77% but increased for S3 by 36.43%. On the device side, early stance generally showed lower extension moments in the passive condition, resulting in an average 153.62% increase in peak extension moments in the powered condition for all subjects.

The frontal plane generally saw mixed results or increased efforts for peak moments and work in the powered condition. On the intact side, peak adduction moment increased for S2 by 29.51% and S3 by 58.50%, but decreased for S1 by 27.07% (see Fig. S8 in Supplemental Material). An identical pattern was observed across subjects on the device side, though significance was only observed in the 33.97% decrease for S1 and 16.96% increase for S3. For the intact side of S2 and S3, the trend continued with peak abduction moment exhibiting an increase of 52.85% for S2 and 25.31% for S3. Abduction moment was generally smaller on the device side than the intact side, especially for S1 and S3, leading to marginal differences in peak values. Most differences in positive or negative work on either side were marginal or not significant (Fig. 7). Only negative work on the intact side exhibited significant changes between non-negligible magnitudes, where S1 decreased by 14.36% and S3 increased by 47.02%.

#### 2) Intact Knee:

Peak extension moments at the intact knee were substantial in early stance but similar between conditions (see Fig. S8 in Supplemental Material). This resulted in mixed and insignificant results, where powered condition values decreased slightly for S1 and increased slightly for S3. Negative knee work, however, consistently increased in the powered condition for all three subjects — by an average of 25.34% — primarily driven by energy absorption during early-stance knee flexion and also in late swing in preparation for heel-strike. Results were also mixed in the frontal plane. Peak knee abduction moments decreased in the powered condition for S1 by 31.02% and S3 by 52.64% but increased for S2 by 46.88%. For peak adduction moments, S1 saw a decrease of 23.43% while S2 and S3 saw increases of 23.41% and 13.81%, respectively.

#### 3) Intact Ankle:

Overall, the intact ankle showed an increase in joint load and energetics across most measures. Peak plantarflexion moment for push-off increased for S1 by 6.12% and S2 by 17.01%. Positive ankle work followed this trend and increased for S1 by 21.75% and S2 by 29.09% (Fig. 7). Negative ankle work had much lower magnitude than positive work but still saw increased values for all three subjects, by an average of 70.24%, mostly from negative power just before push-off. Similarly small in magnitude, peak dorsiflexion moment during early stance increased for S2 by 26.11% and S3 by 44.95%, but decreased for S1 by 27.09%.

#### 4) Total Work:

Level-ground walking is typically a balanced activity in terms of energy generation and absorption. The powered condition had mixed results compared to passive: two subjects increased weighted absolute work (Fig. 7), each through different mechanisms. Subject 3 increased both total positive and negative work by 24.19% and 13.69%, respectively, leading to a 22.01% increase in weighted absolute work. Subject 2 instead decreased total negative work by 17.14% while increasing total positive work by 47.86%. These changes resulted in a 29.21% decrease in weighted absolute work. Subject 1 had no notable changes (Fig. 7).

### D. Stair Descent.

#### 1) Hips:

During stair descent, the powered condition generally reduced intact-side sagittal-plane hip effort in terms of moments and energetics (device-side hip kinetics were excluded due to measurement artifacts). All three subjects reduced their peak flexion moment, by an average of 32.58%. Similarly, positive sagittal-plane hip work decreased for all subjects, though this was only significant for S1 (by 34.38%) and S2 (by 47.42%). Additionally, all subjects exhibited a reduction in negative sagittal-plane hip work by an average of 50.53% on the intact side.

In contrast, compensatory hip activity in the frontal plane had mixed results. The powered condition increased the peak adduction moment on the intact side for S2 by 83.87% and S3 by 24.26% but decreased it for S1 by 23.41%. Frontal-plane hip powers were also generally larger on the intact side than the device side (see Fig. S9 in Supplemental Material). The powered condition increased positive work of two subjects: S2 by 97.58% and S3 by 41.88% on the intact side.

#### 2) Intact Knee:

The powered condition reduced the peak sagittal-plane knee extension moment on the intact side for all three subjects by an average of 12.58%. The difference was much larger during early stance, whereas moments in late stance remained similar between conditions (see Fig. S9 in Supplemental Material). A similar pattern was observed for negative sagittal-plane power; in late stance, power remained similar for two subjects and decreased for one subject, consequently leading to mixed results in negative work: S1 increased effort by 11.41% while S2 decreased by 28.56%. Frontal-plane knee abduction moments followed an identical trend as the sagittal plane, with a reduction for all three subjects by an average of 34.29%.

#### 3) Intact Ankle:

The powered condition increased intact-side ankle loads and energetics for S1 and S2 but reduced these measures for S3. Peak plantarflexion moment increased for S1 by 26.85% and S2 by 20.61%, but decreased for S3 by 27.04%. Due to negative power differences in early stance (see Fig. S9 in Supplemental Material), negative work increased for S1 by 6.18% and S2 by 56.26%, but decreased for S3 by 21.68%. Positive work also increased for S1 and S2 but decreased for S3.

#### 4) Total Work:

**Fig. 8.**
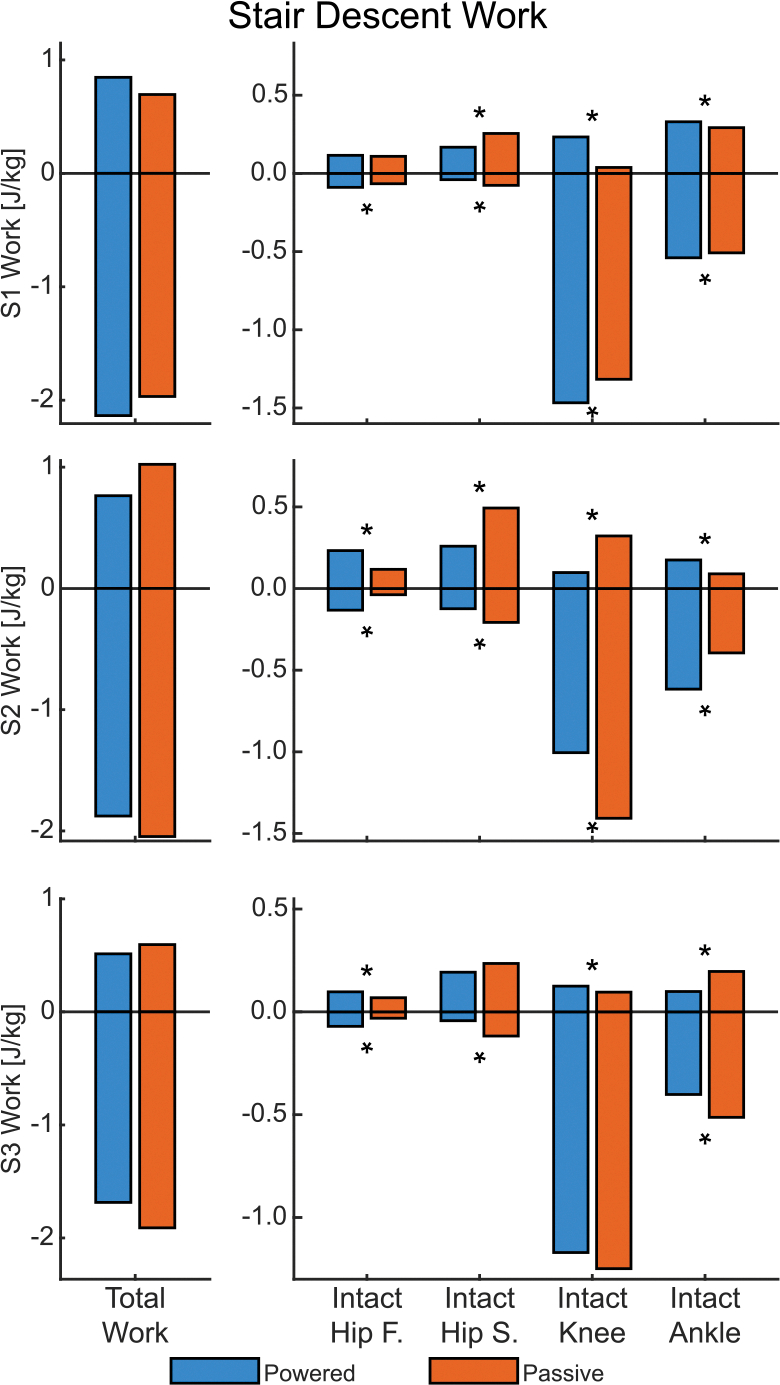
The total biological work and joint components for stair descent. On the left is total work — the sum of the individual joint components on the right. Frontal plane is abbreviated as F and sagittal plane as S. An asterisks (*) denotes significant difference (*α* < 0.05) between the conditions. Significance was not tested for Total Work. Device-side hip is excluded due to force plate measurement artifacts caused by the stair descent strategy in the passive case.

Changes in total work metrics (excluding device-side hip kinetics) during stair descent mostly favored the powered prosthesis (Fig. 8). This condition reduced total positive work for all three subjects by an average of 20.01%. Total negative work decreased by 4.21% and 9.29% for S2 and S3, respectively, leading to a 11.83% and 24.09% reduction in weighted absolute work. While S1 increased total negative work by 9.31%, they still exhibited an 8.52% decrease in weighted absolute work (Fig. 8) due to reduced positive work.

### E. Stair Ascent.

Due to the difference in stair ascent strategy, S2 is not included in the comparisons below, though the values are still reported in Table S-V in the Supplemental Material.

#### 1) Hips:

On the device side, S1 generally decreased sagittal-plane hip loads in the powered condition, while S3 increased loads. On the intact side, S1 decreased peak extension and peak flexion moments by 14.40% and 20.00%, respectively. Positive sagittal-plane power on the intact side was consistently lower throughout stance in the powered condition for S1 (see Fig. S10 in Supplemental Material), leading to positive sagittal-plane work decreasing by 13.32%. Meanwhile S3 increased peak extension and peak flexion moments on the intact side by 25.51% and 28.76%, respectively. The larger early stance extension moment in the powered condition also affected positive power, leading to increased positive sagittal-plane work by 22.34% on the intact side for S3, as joint velocities were comparable between conditions.

The trend largely continued in the frontal plane, with S1 decreasing hip effort and S3 increasing hip effort. On the intact side, S1 decreased peak adduction by 41.16% while S3 increased peak adduction by 32.77%, primarily due to changes in late stance for both subjects. Negative work on the intact side followed an identical trend, decreasing for S1 by 27.17% while increasing for S3 by 27.70%, though mainly due to changes in negative power in early-to-mid stance for both subjects. Device-side adduction moments and negative work tended to be smaller, which can exaggerate relative differences. On the device side, S3 decreased peak adduction moment by 64.50% and negative work by 84.94%. Notably, peak abduction moment increased for S1 by 17.60% and S3 by 55.16% on the intact side, while decreasing for S1 by 44.84% and S3 by 70.66% on the device side.

#### 2) Intact Knee:

While the powered condition did not change the peak sagittal-plane knee extension moment in early stance for S1, this metric decreased for S3 by 52.90%. The latter case coincided with a reduction in positive sagittal-plane knee power (see Fig. S10 in Supplemental Material), resulting in positive work decreasing for S3 by 28.51%. Subject 1 also saw a reduction in positive sagittal-plane knee work by 12.67%, but primarily due to a decrease in power in late stance. In the frontal plane, knee adduction moments decreased by 58.39% for S1 but did not change for S3. Knee abduction moments decreased for both subjects: S1 by 31.79% and S3 by 25.74%.

#### 3) Intact Ankle:

Peak plantarflexion moments had mixed results for the two subjects: the powered condition caused an insignificant increase for S1 but a significant decrease for S3, by 4.05%. This peak, however, occurs during late stance and therefore does not capture the decrease in plantarflexion moment both subjects exhibited during early stance in the powered condition (see Fig. S10 in Supplemental Material). With that, the powered condition decreased positive ankle work for both subjects, by 36.20% for S1 due to decreased positive power through early-to-mid stance, and by 17.54% for S3 due to decreased positive power in late stance.

#### 4) Total Work:

**Fig. 9.**
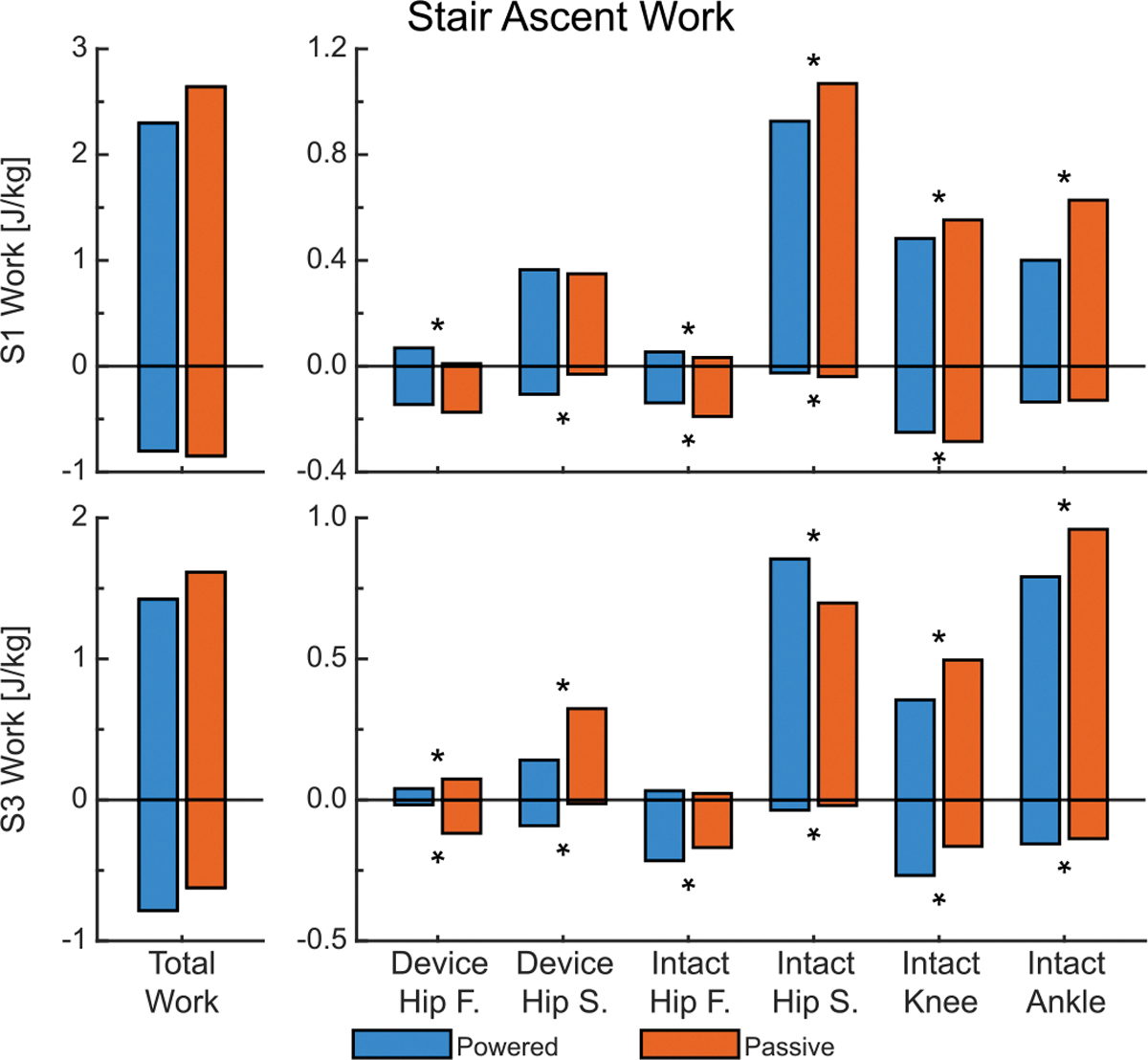
The total biological work and joint components for stair ascent. On the left is total work — the sum of the individual joint components on the right. Frontal plane is abbreviated as F and sagittal plane as S. An asterisks (*) denotes significant difference (*α* < 0.05) between the conditions. Significance was not tested for Total Work. Subject 2 is not shown due to the difference in stair ascent strategies used between conditions.

During stair ascent, both S1 and S3 showed consistent reductions in total joint work metrics with the powered condition (Fig. 9). Both S1 and S3 decreased total positive work by 13.03% and 14.01%, respectively, contributing to reductions in weighted absolute work by 12.56% for S1 and 12.09% for S3. In contrast, total negative work increased for S3 by 26.10%, a breakdown of which can be seen in Fig. 9.

## IV. Discussion

In this case series, we compared amputee joint biomechanics when using a powered knee-ankle prosthesis vs. passive devices during a continuous, sustained sequence of the primary activities of daily life. This work builds on prior studies of the same powered device/controller showing normative able-bodied trends in prosthetic joint kinetics and energetics during walking [26], stair ascent/descent [27], and sit-stand transitions [18], which are largely confirmed in the Supplemental Material. While prior work [21] also studied intact joint biomechanics with a powered knee-ankle prosthesis (the Open-Source Leg using a conventional controller), that study did not consider the same set of activities (e.g., missing sit-stand transitions) over a sustained period of time. Our results indicate that, while not universally beneficial, our powered device tended to reduce intact joint effort, quantified using moments and work. The sustained and repetitive nature of the experiment suggests that the benefits shown may translate to real-world ambulation and hold true even in a fatigued state.

### A. Interpretation of Results.

In this case series, the powered device generally benefited total work metrics for four of the five activities. Weighted absolute work (a proxy for overall effort) decreased in at least half of the activities, where the specific activities varied by subject. Notably, all three subjects reduced weighted absolute work during stand-to-sit and stair descent. In sit-to-stand and stair ascent, two subjects decreased weighted absolute work. The exception, S2, exhibited a 2.89% increase during sit-to-stand, and his step-by-step ascent strategy in the passive condition prevented direct comparison to the powered condition.

Our observed reductions in total work metrics generally improved upon the changes observed in [21], likely due to differences in powered prosthesis hardware and/or control. During step-over-step stair ascent, our average 12.32% reduction in weighted absolute work contrasts with the 0.81% *increase* in [21], and our 13.52% reduction in total positive work contrasts with the 2.17% *increase* in [21]. While we also observed reductions in positive work during sit-to-stand (two subjects, 32.11% and 27.78%), this task was not studied in [21]. Our observed 14.82% reduction in weighted absolute work during stair descent was similar to the 12.19% reduction in [21]. We also saw decreases in total negative work for stand-to-sit, which was not studied in [21]. Moreover, the activities tested in [21] involved fewer repetitions (10 laps vs. 18–110 in our study), so it is unclear if their results would still hold under sustained use.

Walking had mixed results in our case series. The powered condition increased peak hip extension moments during early stance for all three subjects, leading to inconsistent increases in positive hip work. Meanwhile, decreased peak hip flexion moments during the stance-to-swing transition led to decreased negative hip work on both sides for all subjects. The powered device likely reduced the hip flexion moment because its powered ankle propelled itself into swing, relieving the pull-off requirements of the user’s hips, which is consistent with previous observations with powered prostheses [19], [34]. The intact ankle, however, saw an increase in both positive and negative peak powers and work. Additionally, the intact ankle provided higher peak plantar- and dorsiflexion moments for the majority of comparisons (4 out of 6). This, along with the mixed total work results, could suggest that work was redistributed from the hips to the ankles during walking with the powered device, which weighs more than 1.5 times that of the heaviest passive device in this study. The powered device appears most beneficial for more demanding activities requiring net changes in work, whereas passive devices are well optimized for level-ground walking, which requires close to net zero work when adding both the knee and ankle [35].

Overall, our results suggest that a powered device has the potential to help reduce hip moments and effort during stair ambulation. We observed a common compensatory strategy on the device side during step-over-step stair ascent with the passive device: extending the knee and abducting the hip to place the foot on the next step. The powered device allowed the subjects to reduce their device-side peak hip abduction moment, suggesting less circumduction despite the greater device weight. This is likely due to the powered device flexing the knee and dorsiflexing the ankle for toe clearance, therefore removing the need for abduction. The increase in intact-side peak hip abduction moment (in swing) could suggest greater trust in the device (in stance), since more biomimetic weight loading on the device leg (as seen in Figs. S4 and S5) would allow for more biomimetic circumduction of the intact leg in swing. During stair descent, the increased peak hip adduction moments brought two subjects closer to biomimetic values and may again reflect greater trust in the device, as they appeared to descend more deliberately rather than with the controlled “falling” strategy typically used with a passive device. The sagittal plane saw reduced peak hip flexion moments in all subjects on the intact side, resulting in reduced positive hip work in two of the three subjects. These stair descent results match those with the Open Source Leg in [21].

Although not the focus of this study, there were some notable observations with asymmetry in the hips. In both conditions, subjects generally relied more on their intact side than the device side for most activities. Nearly all subjects presented this bias in sagittal-plane positive work for activities predominately requiring energy generation (17 out of 18 comparisons), and all subjects favored the intact side for negative work during stand-to-sit. This asymmetry in the passive condition is expected due to the lack of device-side power, but its persistence in the powered condition is unexpected given the power generation at the motorized joints. A similar pattern has been reported in stair ambulation during stance phase, where power magnitudes dominate [21]. Sagittal-plane hip moments also showed asymmetry in both conditions, most notably during sit-to-stand, where two of the three subjects exhibited substantial differences in peak extension moments between sides, regardless of device condition. These asymmetries have been observed before with this powered prosthesis [36], but appeared more pronounced here, likely due to the sustained, repetitive, and timed nature of the trials where subjects quickly transitioned from sit-to-stand into walking rather than prioritizing symmetrical motion. Stand-to-sit saw similar asymmetries for the same two subjects for likely the same reason. For the two subjects that used a step-over-step strategy in stair ascent, peak extension moment and sagittal-plane positive power were also considerably asymmetric in both conditions. This could be due to the stepping strategy during this activity, as discussed later in the Limitations and Future Work section. For more analysis on symmetry in this study, please refer to Supplemental Material Section IV.

The knee was the intact joint that benefited the most from the powered condition. All three subjects exhibited a significant decrease in peak sagittal-plane extension moment at the knee in three of the activities — sit-to-stand, stand-to-sit, and stair descent. During step-over-step stair ascent, one of the two applicable subjects decreased their peak knee extension moment, and the other subject was a null result. Both of these subjects experienced a significant reduction in positive sagittal-plane knee work, a pattern that was also observed during sit-to-stand. Further, frontal-plane knee abduction moments were decreased for at least two subjects in all five activities. These observed reductions suggest that powered prostheses have the potential to help reduce the prevalence of intact-side knee pain and osteoarthritis in transfemoral amputees [7], [8].

While biomechanical outcomes were highly individualized, the powered device enabled consistent performance over time and elicited strong user acceptance. For each trial, we did not observe meaningful variation in our metrics over time, though different metrics may reveal the effects of fatigue. Overall, S1 experienced more biomechanical benefits than the other two subjects, though all subjects reported liking the powered device. Subject 3 reported that stair ascent was “the closest I’ve felt since having two legs”, and that “step-over-step is very good for my mental health.”

### B. Limitations and Future Work.

A primary limitation of this work concerns the generalizability of the findings due to the small and homogeneous sample of above-knee amputee subjects (though we had a large number of within-subject samples). The device’s height constraints excluded shorter individuals, which in practice restricted enrollment to male subjects. The protocol’s high physical demands also limited participation to those with higher mobility and fitness level, which contributed to at least one of the original five participants withdrawing before completing the multi-day protocol. Future work could implement similar adaptable control techniques on a smaller robotic prosthesis like the Open Source Leg [37] or the Utah Leg [22] to enable a larger, more diverse cohort, including individuals with lower mobility levels and female subjects. Such studies may reveal different results than found with traditional finite-state machine controllers in [14] and [21].

The study also had limitations regarding the available force plate kinetics data. No force plates were embedded in the ramp portion of the circuit, so ramp activities were excluded from the analysis. Moreover, only a few usable strides per lap were captured for level-ground walking and stair ambulation. Subjects were not instructed to target specific foot placements, so they sometimes stepped with both feet on a single plate or partially off a plate during level walking, leading to exclusion of those strides. During stair descent with the passive device, data quality was diminished by frequent edge loading on the protective rail at the front of the stair force plates — which is why the device-side hip was not analyzed for this task. Further, handrail usage was not measured during the experiment, and modulated only through frequent reminders to “not overly rely on the handrails.” Future work should consider ways to increase the quantity and quality of kinetic data, including an improved protective rail design in the instrumented stairs to accommodate edge loading. Finally, this study only analyzed steady-state strides, leaving transitions between activities to future work.

## V. Conclusion

This case series suggests that a powered knee-ankle prosthesis, when utilized across a sustained, continuous circuit of daily activities, has the potential to mitigate detrimental loading on the intact joints. For the three subjects, the observed reductions in peak moments and total work metrics across daily activities — though less pronounced for walking — directly address the compensatory overuse of intact joints that often leads to joint degeneration in above-knee prosthesis users. These exploratory results motivate future studies on the potential benefits of powered knee-ankle prostheses on long-term musculoskeletal health and functional well-being for users.

## Supplementary Material

supp1-3659043

This article has supplementary downloadable material available at https://doi.org/10.1109/TNSRE.2026.3659043, provided by the authors.

## Figures and Tables

**TABLE I:** Subject Information and Measurements

	Sex	Age	Years Since Amputation	Amputation Side	Height [m]	Mass [kg]	K-Level	Passive Knee & Ankle/Foot	Preferred Stair Ascent Strategy
Subject 1 (S1)	M	23	9.5	Right	1.75	81.85	K4	Ottobock Genium & Ottobock Triton	Step-over-step
Subject 2 (S2)	M	29	28	Left	1.85	121.25	K4	Ottobock C-Leg & Ottobock Trias	Step-by-step
Subject 3 (S3)	M	20	2	Right	1.85	73.90	K4	Proteor Quattro & Proteor ShockWave	Step-over-step
