# Supplementary material for: Effects of a Powered Knee–Ankle Prosthesis on Intact Joint Biomechanics Across Sustained Activities of Daily Life: A Case Series
Source: IEEE Trans Neural Syst Rehabil Eng. Author manuscript; Available in PMC 2026 Mar 25. (PMC13015786; doi:10.1109/TNSRE.2026.3659043)
Supplement: supp1-3659043 [file NIHMS2145357-supplement-supp1-3659043.pdf]

Supplemental Material for:

Effects of a Powered Knee-Ankle Prosthesis on Intact Joint Biomechanics Across Sustained Activities of Daily Life: A Case Series

Emily G. Keller, Curt A. Laubscher, and Robert D. Gregg

Corresponding author: Robert D. Gregg, rdgregg@umich.edu

DOI:

I Supplemental Methods . . . 2
I-A Modified Plug-in Gait . . . 2
I-A1 Knee and Ankle Axes . . . 2
I-A2 Pelvis Mediolateral Axis . . . 2
I-A3 Inertial Properties . . . 2
I-B Linear Model for Statistical Analysis . . . 3

II Time Series Figures . . . 4

III Joint Kinetics Tables . . . 12

IV Symmetry Analysis . . . 18

References . . . 20

I. Supplemental Methods

A. *Modified Plug-in Gait*

This section describes the modifications that were made to Vicon's Plug-in Gait. Modifications were made to the standard Vicon Plug-in Gait in order to more directly determine the knee and ankle axes and to provide alternative methods to determine the axis of the pelvis in the event of marker occlusion during sitting and standing. Modifications were also made to the inertial properties of the segments to accurately reflect that of the prosthetic devices and residual limb. Each change is described in detail in the sections below.

1) Knee and Ankle Axes: Vicon describes the anatomical ankle and knee axes as between the medial and lateral markers at the joint. The joint center, referred to as the origin, is located at the midpoint between these two markers. In practice, however, the medial marker is not used, and the thigh and tibia markers are used as proxies.

In an effort to more directly determine the joint center and therefore the sagittal joint axis, a modified version of Plug-in Gait was implemented that defines the origin as the midpoint between the medial and lateral markers. This directly uses the medial marker to determine the sagittal joint axis — since Vicon describes the anatomical joint axis relative to it — as opposed to indirectly approximating it with the thigh and tibia markers.

Due to concerns about tissue movement of the thigh and shank during the highly dynamic tests performed in this study, the modified Plug-in Gait logic was used as the primary logic. If the medial marker was not available after gap filling, Vicon's default logic was used.

2) Pelvis Mediolateral Axis: The circuit required that the subject be seated at the beginning, middle, and end of each lap. When the subject is lowering, they would lean forward and push their hips back, often causing occlusion of the anterior superior iliac spine (ASIS) markers. Depending on the subject, the occlusion could continue while seated.

Vicon's Plug-in Gait defines the mediolateral axis of the pelvis between the two ASIS markers, with the origin of the pelvis at their midpoint. If an ASIS marker was missing, the pelvis could not be made and therefore the rest of the skeleton would not be made. Due to the pelvis being the foundation of the skeleton and the frequent occlusion of the ASIS markers when sitting, backup logic was made to help when the ASIS markers could not be gap filled.

Extra markers were placed on the subject's greater trochanters (GT) to provide another set of markers that could be used to define the mediolateral axis of the pelvis. These markers are not likely to be occluded given that they are on the lateral sides of the subject and therefore always visible through sitting and being seated. The line between the GT markers and the line between the ASIS markers run parallel so the axis defined would be the same. To locate the origin of the pelvis the inter-ASIS distance was utilized — a measurement of the subject that is taken during calibration. The origin is placed half the inter-ASIS distance along the pelvis mediolateral axis from the remaining ASIS marker. It is a limitation of this backup logic that at least one ASIS marker must remain, otherwise the origin cannot be calculated, even if the axis can.

3) Inertial Properties: Vicon's Plug-in Gait assumes able-bodied proportions for length, mass, and center of mass of the limb segments. For this study, this assumption fails on two accounts: 1) the device side limb does not have able-bodied proportions, and 2) the proportions are dependent on the prosthetic device used. In order to get more accurate kinetics of our subjects, the inertial properties of the device side limb were changed based on the device used.

The mass and centers of mass of the powered device's segments were estimated using the CAD model of the device. The mass of the passive devices was found from the product descriptions and the centers of mass were estimated as halfway along the length of the device (not including any pylon length), and centered in the transverse cross-section.

The thigh was modeled using the residual limb length and components of the powered device that are part of the thigh. A scaling fraction was calculated:

$$fraction = residual\_length/(total\_height \cdot 0.245)$$

where 0.245 is the anatomical fraction of the thigh relative to total height from [1], and the fraction that Vicon uses natively. The fraction was used to scale the mass and center of mass of the residual limb. When modeling for the passive devices, the thigh was modeled using only the fraction of the residual thigh, as it could not be known what mass of the passive device should be attributed to the thigh.

The shank masses were estimated using the weight of the passive knee or the components of the powered device attributed to the shank. The feet masses were estimated using the passive feet or the components of the powered device attributed to the foot and the foot attached to it.

B. Linear Model for Statistical Analysis

A linear model was chosen because it accounts for the repeated structure of the data — multiple strides within an activity per lap and multiple laps per condition — and allows each subject to be modeled individually, with their own distinct response to the device condition. This approach preserves all available samples while appropriately modeling their dependence within each subject. The model is as follows:

$$\begin{aligned} Value \sim -1 &+ (1 + Side * Condition) : SubjectOne \\ &+ (1 + Side * Condition) : SubjectTwo \\ &+ (1 + Side * Condition) : SubjectThree \end{aligned}$$

where Side and Condition are variables coded using simple reference coding: device relative to intact for Side, and powered relative to passive for Condition. Side was included as an interaction term so that each side was allowed to react differently to Condition. It was clear from preliminary exploration that this would be necessary. Subjects are represented as separate dummy-coded binary indicator variables, and the model omits an overall intercept, allowing for each subject to be estimated directly (cell means coding). This parameterization ensures that all comparisons are made within subjects, and no explicit between-subjects effects are tested. The chosen model allows each side of each subject to be grouped independently and compared between conditions without doing any comparisons between subjects (i.e., six independent comparisons). Given that $N = 3$ for this study, we do not have enough subjects to make population claims, so between subject comparisons were not done.

Each run of the model had data for all subjects, all sides, all conditions, for a given joint metric within an activity. Hence, this is why joint and activity do not appear in the model as they are already accounted for in the data passed in Value. We did not do any *statistical* analysis across activities or across joints.

Bold text in the tables in Supplemental Section III indicates comparisons that showed statistically significant differences with a significance level of 0.05, even if it was not presented in the main text.

II. Time Series Figures

The following graphs show the kinematics and kinetics of each subject. Figures S1–S5 show the ground reaction forces (GRF) in the vertical and the anterior/posterior directions for each subject and each activity. Figures S6–S10 show the joint angles, moments, and powers for each side of each subject and each activity. Able-bodied frontal plane knee metrics are not shown due to the scarcity of this data in published data sets.

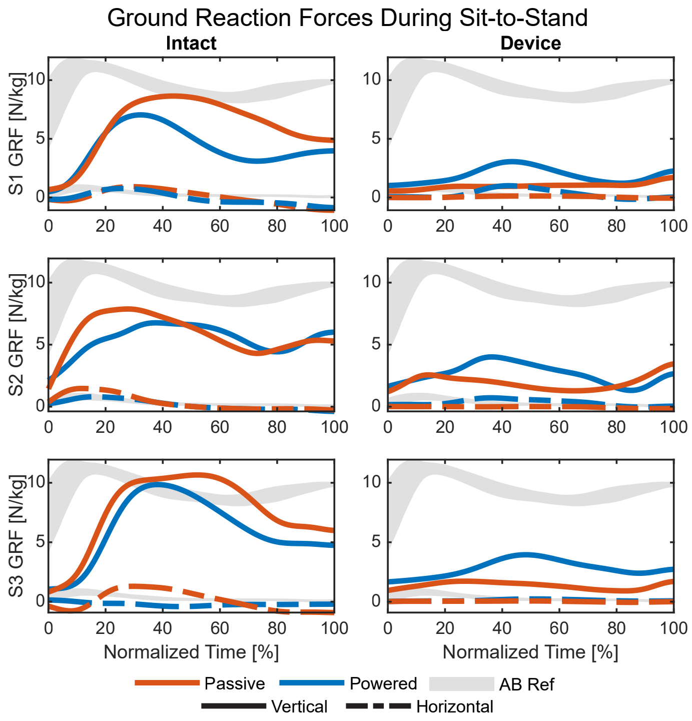

Fig. S1. Average ground reaction forces during sit-to-stand for each subject (S1, S2, and S3). The powered condition is shown in blue and the passive condition in orange. A reference for ±1 standard deviation of normative able-bodied trajectories is shown shaded in gray from [2]. The vertical ground reaction force is shown as a solid line and the anterior/posterior ground reaction force as a dashed line.

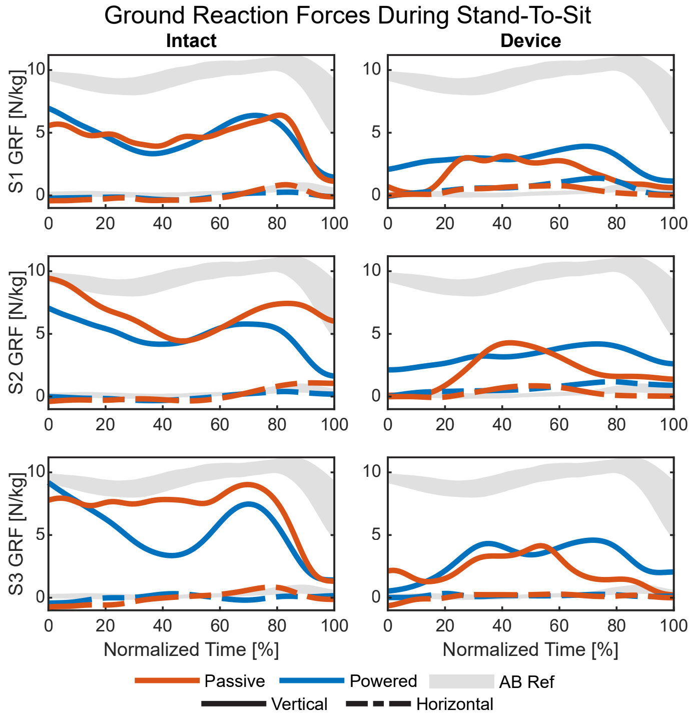

Fig. S2. Average ground reaction forces during stand-to-sit for each subject (S1, S2, and S3). The powered condition is shown in blue and the passive condition in orange. A reference for ±1 standard deviation of normative able-bodied (AB) trajectories is shown shaded in gray from [2]. The vertical ground reaction force is shown as a solid line and the anterior/posterior ground reaction force as a dashed line.

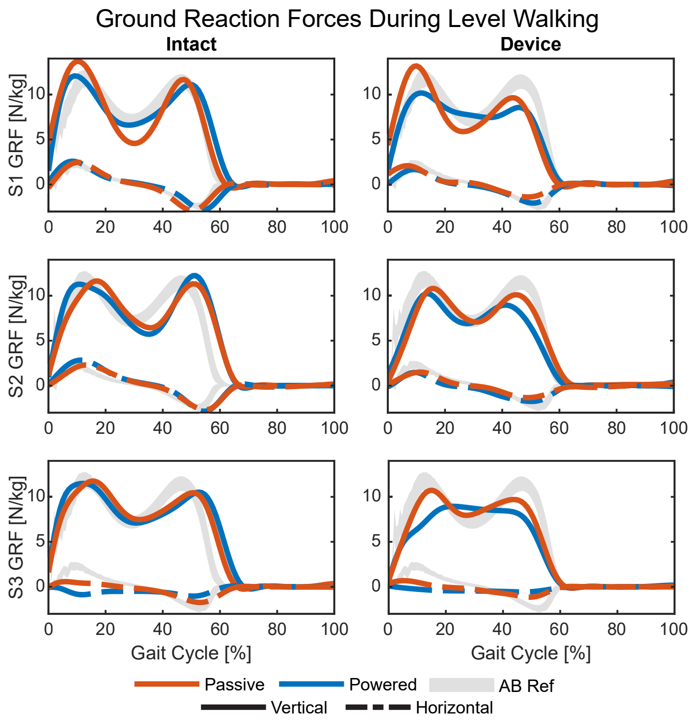

Fig. S3. Average ground reaction forces during level walking for each subject (S1, S2, and S3). The powered condition is shown in blue and the passive condition in orange. A reference for ±1 standard deviation of normative able-bodied (AB) trajectories is shown shaded in gray from [3]. The vertical ground reaction force is shown as a solid line and the anterior/posterior ground reaction force as a dashed line.

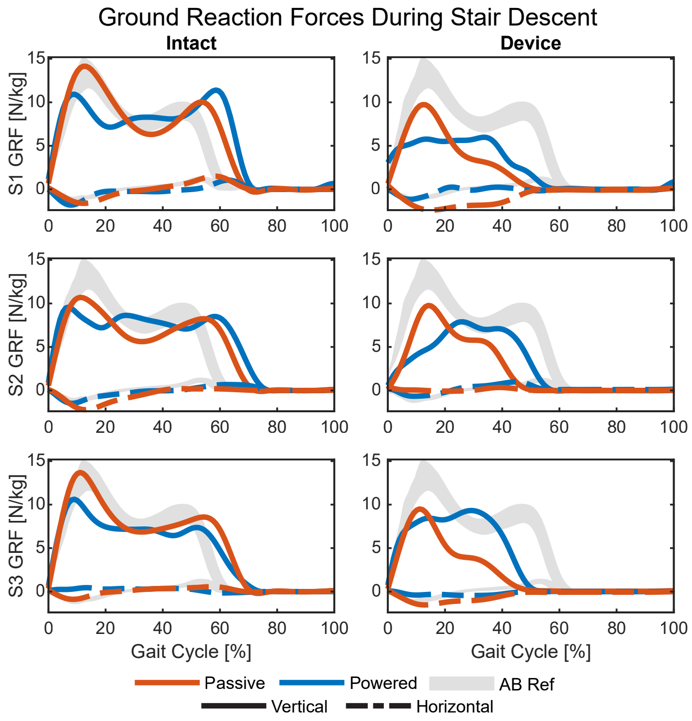

Fig. S4. Average ground reaction forces during stair descent for each subject (S1, S2, and S3). The powered condition is shown in blue and the passive condition in orange. A reference for ±1 standard deviation of normative able-bodied (AB) trajectories is shown shaded in gray from [3]. The vertical ground reaction force is shown as a solid line and the anterior/posterior ground reaction force as a dashed line. The GRF for the device-side passive condition is lower in the second half of stance due to the foot rolling over the protective rail of the stair and not the force plate.

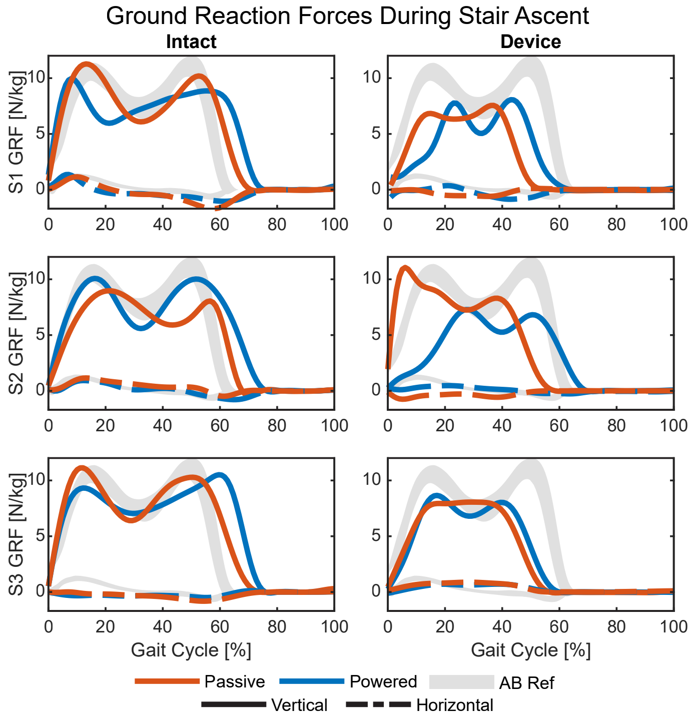

Fig. S5. Average ground reaction forces during stair ascent for each subject (S1, S2, and S3). The powered condition is shown in blue and the passive condition in orange. A reference for ±1 standard deviation of normative able-bodied (AB) trajectories is shown shaded in gray from [3]. The vertical ground reaction force is shown as a solid line and the anterior/posterior ground reaction force as a dashed line. Subjects 1 and 3 went step-over-step for both conditions while Subject 2 went step-by-step for the passive condition.

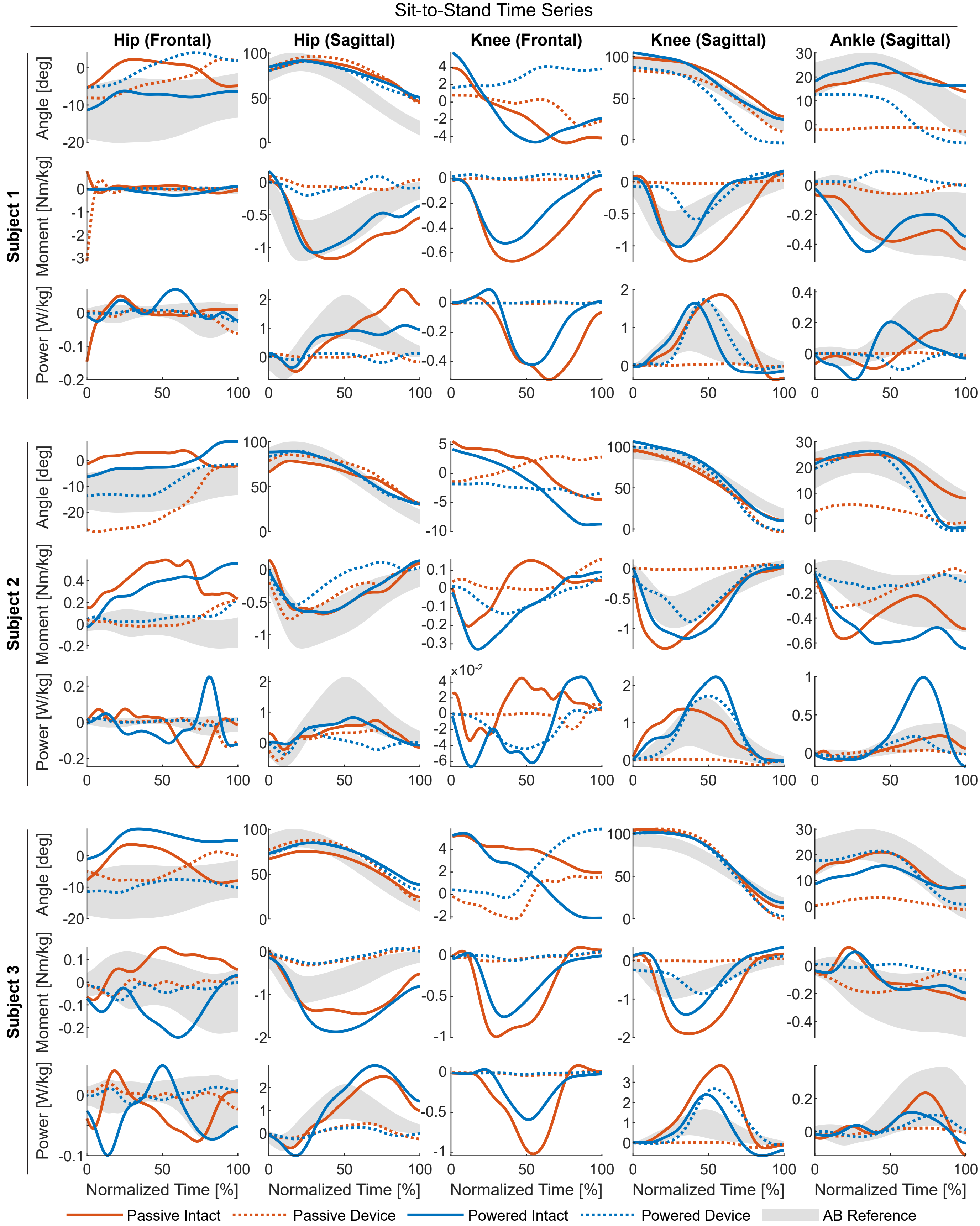

Fig. S6. Average sit-to-stand data for each subject. The powered condition is shown in blue and the passive condition in orange. A reference for ±1 standard deviation of normative able-bodied (AB) trajectories is shown shaded in gray from [4]. The intact side is shown as a solid line and the device side as a dotted line. A positive angle indicates dorsi/flexion or adduction and a positive moment indicates a flexion or adduction moment.

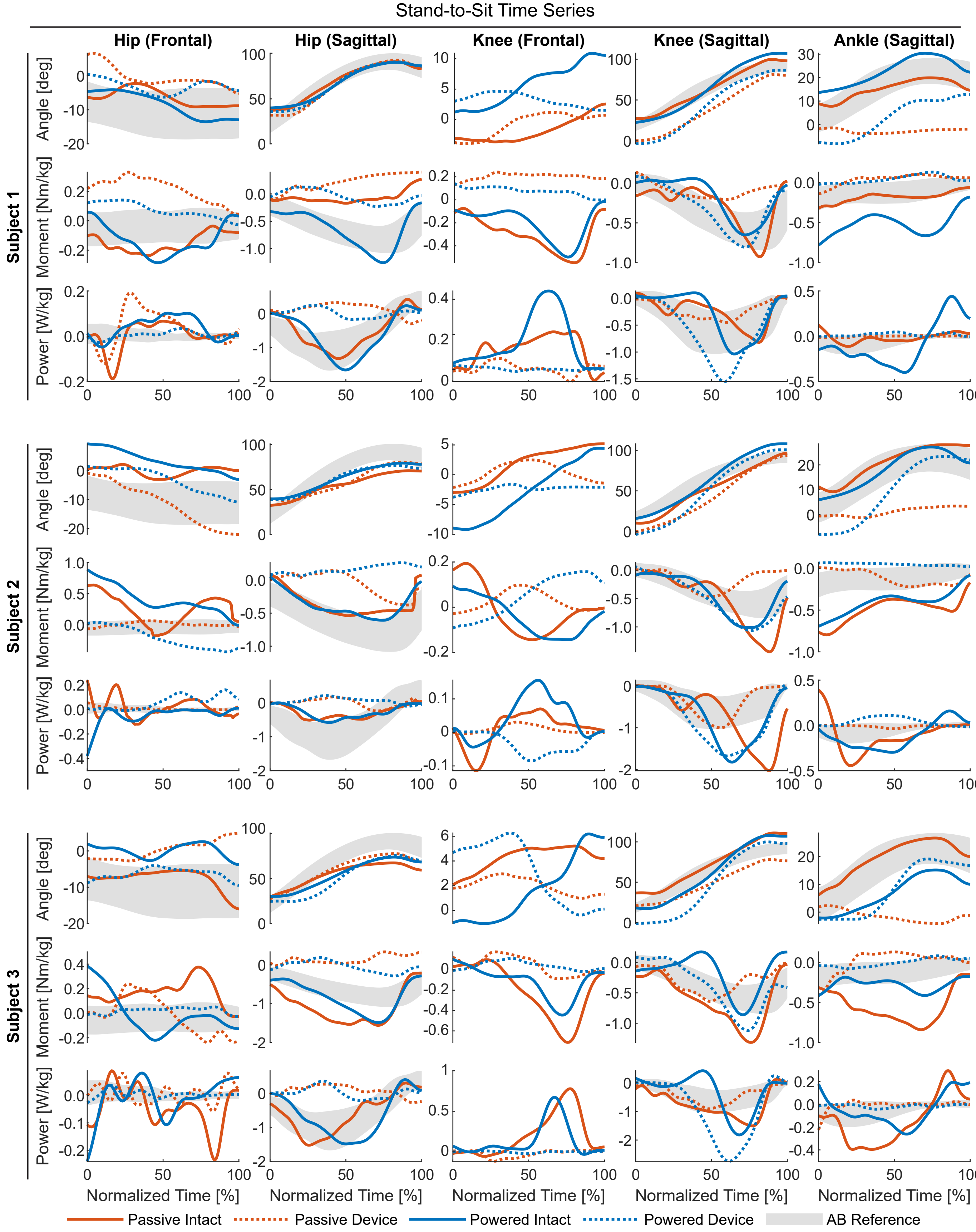

Fig. S7. Average stand-to-sit data for each subject. The powered condition is shown in blue and the passive condition in orange. A reference for ±1 standard deviation of normative able-bodied (AB) trajectories is shown shaded in gray from [4]. The intact side is shown as a solid line and the device side as a dotted line. A positive angle indicates dorsi/flexion or adduction and a positive moment indicates a flexion or adduction moment.

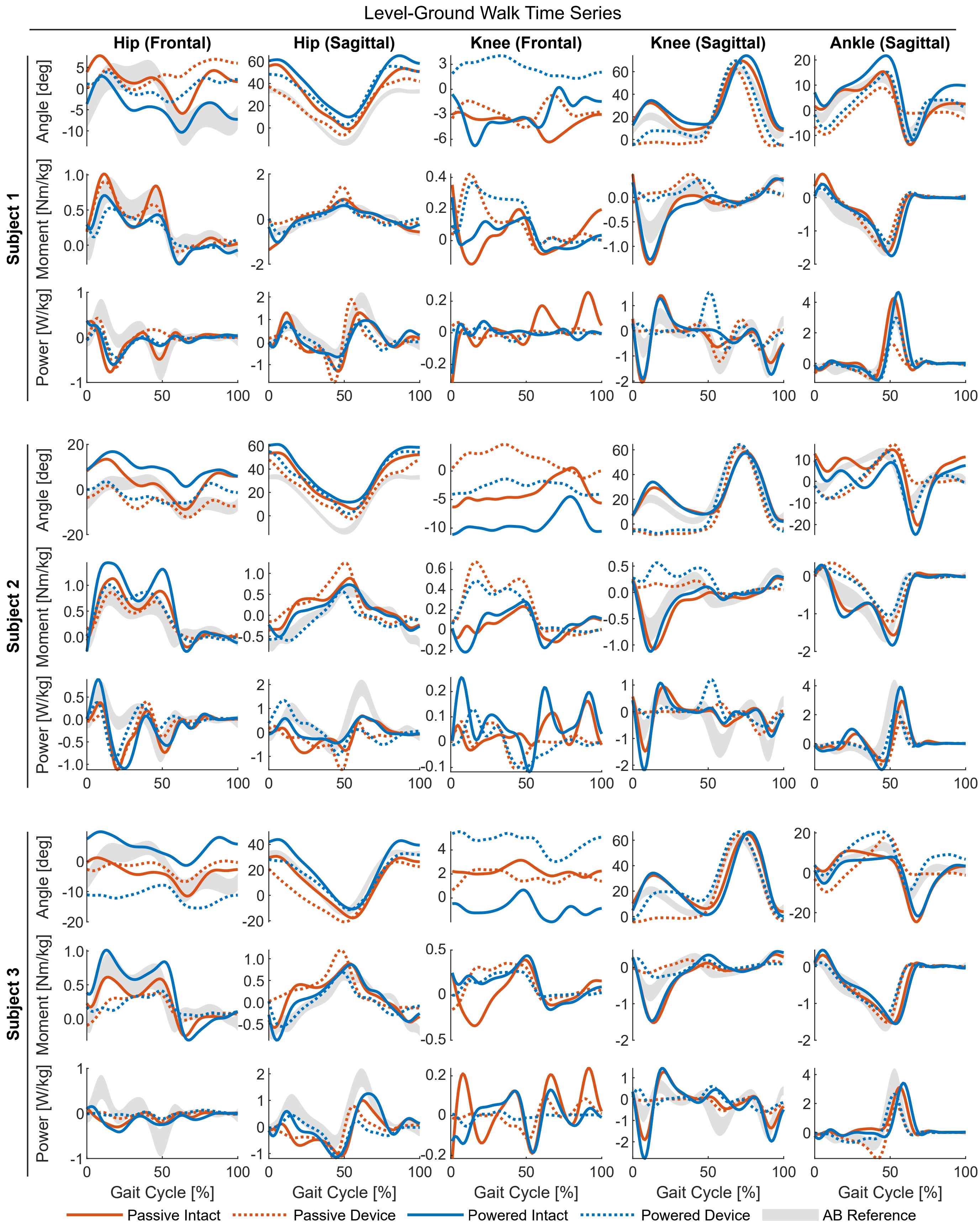

Fig. S8. Average level-ground walking data for each subject. The powered condition is shown in blue and the passive condition in orange. A reference for ±1 standard deviation of normative able-bodied (AB) trajectories is shown shaded in gray from [3]. The intact side is shown as a solid line and the device side as a dotted line. A positive angle indicates dorsi/flexion or adduction and a positive moment indicates a flexion or adduction moment.

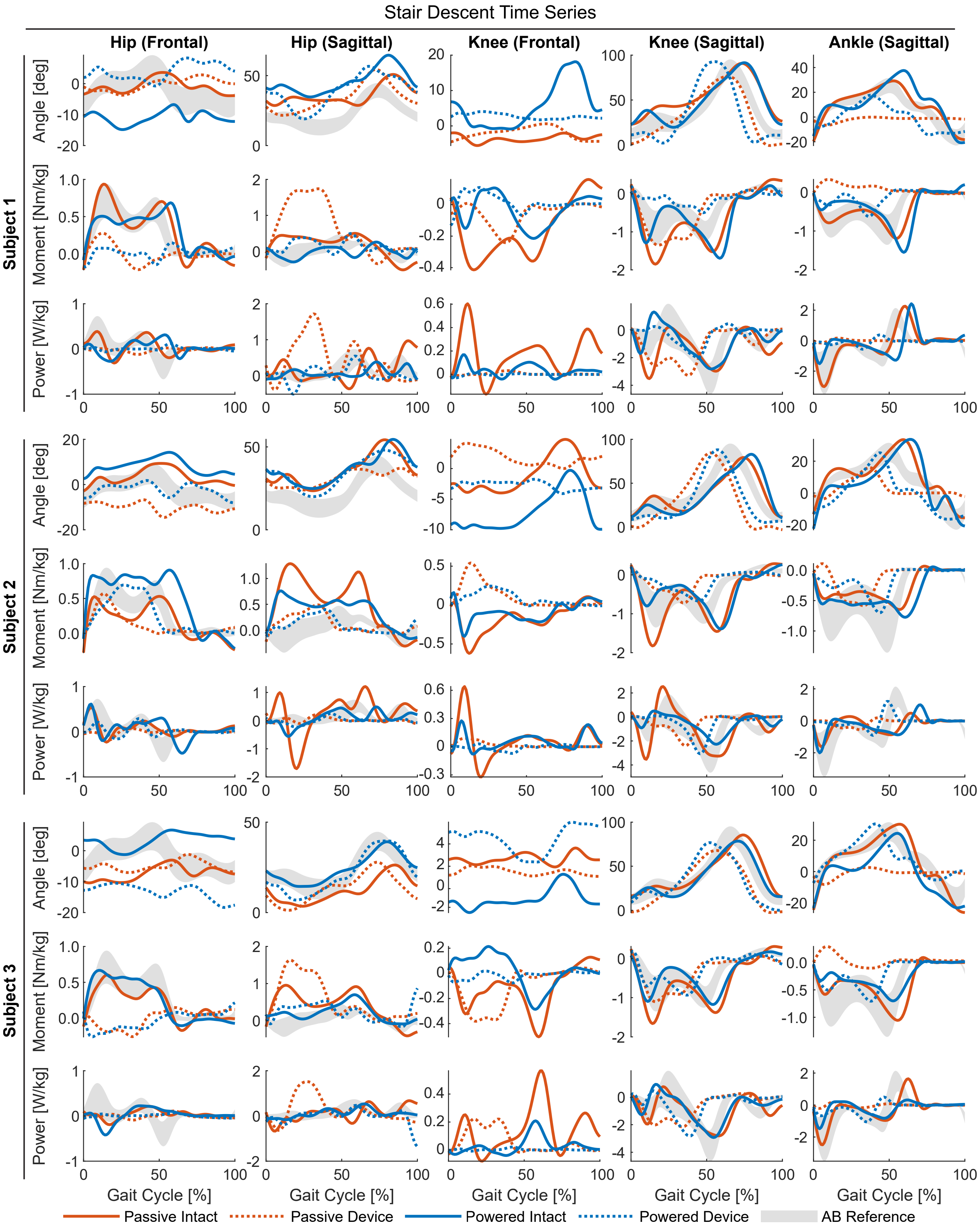

Fig. S9. Average stair descent data for each subject. The powered condition is shown in blue and the passive condition in orange. A reference for ±1 standard deviation of normative able-bodied (AB) trajectories is shown shaded in gray from [3]. The intact side is shown as a solid line and the device side as a dotted line. A positive angle indicates dorsi/flexion or adduction and a positive moment indicates a flexion or adduction moment.

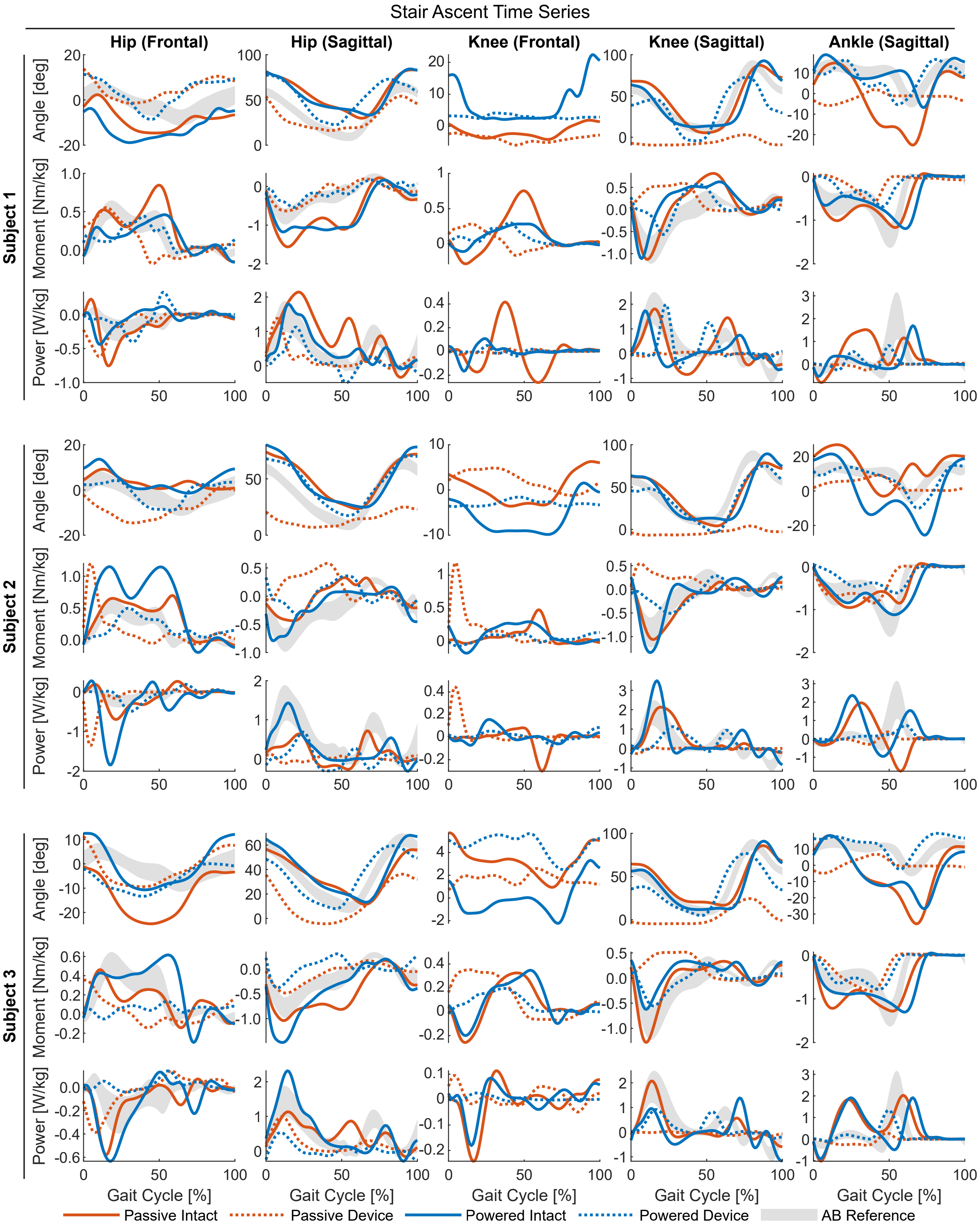

Fig. S10. Average stair ascent data for each subject. The powered condition is shown in blue and the passive condition in orange. A reference for ±1 standard deviation of normative able-bodied (AB) trajectories is shown shaded in gray from [3]. The intact side is shown as a solid line and the device side as a dotted line. A positive angle indicates dorsi/flexion or adduction and a positive moment indicates a flexion or adduction moment. Subjects 1 and 3 went step-over-step for both conditions while Subject 2 went step-by-step for the passive condition.

III. Joint Kinetics Tables

Tables S-I–S-V show kinetic metrics by activity. Data is presented as the mean ± standard deviation rounded to two decimal places for each plane of each joint considered. Total works are not shown with standard deviations. The frontal plane of the knee is shown only for moments, and not for power or work due to the low range of motion and angular velocity of this degree of freedom.

TABLE S-I
SIT-TO-STAND KINETICS

				Subject 1		Subject 2		Subject 3	
				Powered	Passive	Powered	Passive	Powered	Passive
GRF [N/kg]	Device Side		Peak Vertical	**3.23 ± 0.14**	**1.70 ± 0.26**	4.04 ± 0.15	3.52 ± 0.74	**4.05 ± 0.37**	**1.93 ± 0.28**
			Peak Propulsion	**-0.35 ± 0.10**	**-0.09 ± 0.03**	**-0.07 ± 0.03**	**-0.21 ± 0.06**	-0.05 ± 0.03	-0.10 ± 0.06
			Peak Braking	**1.10 ± 0.04**	**0.15 ± 0.07**	**0.76 ± 0.05**	**0.05 ± 0.03**	**0.22 ± 0.06**	**0.11 ± 0.06**
	Intact Side		Peak Vertical	**7.18 ± 1.74**	**8.83 ± 1.52**	**6.92 ± 0.38**	**8.23 ± 0.54**	**9.95 ± 0.52**	**10.80 ± 0.41**
			Peak Propulsion	**-0.87 ± 0.27**	**-1.11 ± 0.32**	-0.39 ± 0.15	-0.33 ± 0.14	**-0.48 ± 0.12**	**-1.07 ± 0.19**
			Peak Braking	**0.79 ± 0.13**	**0.88 ± 0.20**	**0.90 ± 0.12**	**1.57 ± 0.19**	**0.16 ± 0.06**	**1.35 ± 0.15**
Moment [Nm/kg]	Device	Hip (Frontal)	Peak Abduction	**-0.03 ± 0.01**	**-3.14 ± 0.00**	-0.03 ± 0.02	-0.05 ± 0.03	-0.09 ± 0.02	-0.08 ± 0.02
			Peak Adduction	**0.09 ± 0.03**	**0.39 ± 0.00**	0.22 ± 0.04	0.22 ± 0.08	0.03 ± 0.02	0.05 ± 0.03
		Hip (Sagittal)	Peak Extension	-0.22 ± 0.03	-0.16 ± 0.15	**-0.61 ± 0.07**	**-0.79 ± 0.11**	-0.30 ± 0.06	-0.36 ± 0.13
			Peak Flexion	**0.17 ± 0.03**	**0.11 ± 0.02**	0.12 ± 0.02	0.10 ± 0.06	0.12 ± 0.05	0.13 ± 0.07
	Intact	Hip (Frontal)	Peak Abduction	-0.32 ± 0.15	-0.33 ± 0.20	-0.05 ± 0.03	-0.08 ± 0.09	**-0.30 ± 0.13**	**-0.10 ± 0.04**
			Peak Adduction	0.15 ± 0.06	0.17 ± 0.11	0.57 ± 0.09	0.57 ± 0.16	0.17 ± 0.17	0.21 ± 0.07
		Hip (Sagittal)	Peak Extension	**-1.11 ± 0.35**	**-1.25 ± 0.32**	-0.72 ± 0.10	-0.76 ± 0.12	**-1.96 ± 0.15**	**-1.55 ± 0.15**
			Peak Flexion	**0.18 ± 0.11**	**0.11 ± 0.06**	0.20 ± 0.09	0.26 ± 0.02	**0.10 ± 0.00**	**0.32 ± 0.05**
		Knee (Frontal)	Peak Abduction	**-0.54 ± 0.11**	**-0.70 ± 0.13**	**-0.37 ± 0.06**	**-0.23 ± 0.10**	**-0.78 ± 0.14**	**-1.02 ± 0.10**
			Peak Adduction	0.06 ± 0.04	0.04 ± 0.03	**0.11 ± 0.04**	**0.20 ± 0.10**	**0.11 ± 0.07**	**0.14 ± 0.05**
		Knee (Sagittal)	Peak Extension	**-1.05 ± 0.34**	**-1.26 ± 0.30**	**-1.19 ± 0.06**	**-1.42 ± 0.07**	**-1.44 ± 0.09**	**-1.92 ± 0.04**
			Peak Flexion	0.19 ± 0.06	0.23 ± 0.12	0.08 ± 0.05	0.05 ± 0.03	**0.38 ± 0.13**	**0.25 ± 0.13**
		Ankle (sagittal)	Peak Plantarflexion	-0.50 ± 0.16	-0.50 ± 0.16	-0.69 ± 0.05	-0.61 ± 0.05	**-0.22 ± 0.07**	**-0.32 ± 0.19**
			Peak Dorsiflexion	**0.26 ± 0.51**	**0.09 ± 0.06**	0.04 ± 0.00	0.02 ± 0.03	0.12 ± 0.07	0.16 ± 0.10
Power [W/kg]	Device	Hip (Frontal)	Peak Negative	-0.03 ± 0.01	-0.07 ± 0.02	**-0.03 ± 0.01**	**-0.15 ± 0.07**	-0.02 ± 0.01	-0.02 ± 0.01
			Peak Positive	0.02 ± 0.01	0.02 ± 0.01	0.04 ± 0.03	0.05 ± 0.03	0.02 ± 0.01	0.03 ± 0.02
		Hip (Sagittal)	Peak Negative	-0.02 ± 0.09	-0.22 ± 0.09	**-0.51 ± 0.18**	**-0.79 ± 0.10**	-0.36 ± 0.12	-0.31 ± 0.13
			Peak Positive	0.20 ± 0.06	0.18 ± 0.12	0.56 ± 0.18	0.56 ± 0.23	0.27 ± 0.12	0.38 ± 0.17
	Intact	Hip (Frontal)	Peak Negative	-0.08 ± 0.03	-0.06 ± 0.02	**-0.26 ± 0.09**	**-0.31 ± 0.16**	-0.16 ± 0.06	-0.13 ± 0.05
			Peak Positive	0.10 ± 0.05	0.09 ± 0.04	**0.31 ± 0.11**	**0.12 ± 0.03**	0.05 ± 0.03	0.06 ± 0.04
		Hip (Sagittal)	Peak Negative	**-0.43 ± 0.24**	**-0.57 ± 0.38**	-0.22 ± 0.12	-0.34 ± 0.15	**-1.11 ± 0.22**	**-0.72 ± 0.09**
			Peak Positive	**1.32 ± 0.55**	**2.65 ± 0.66**	0.94 ± 0.35	0.95 ± 0.30	**3.19 ± 0.50**	**2.71 ± 0.58**
		Knee (Sagittal)	Peak Negative	-0.40 ± 0.27	-0.46 ± 0.27	-0.08 ± 0.07	-0.11 ± 0.14	**-0.74 ± 0.40**	**-0.32 ± 0.25**
			Peak Positive	1.88 ± 0.69	2.10 ± 0.67	**2.31 ± 0.30**	**1.50 ± 0.21**	**2.65 ± 0.47**	**4.06 ± 0.53**
		Ankle (Sagittal)	Peak Negative	**-0.19 ± 0.06**	**-0.12 ± 0.07**	**-0.22 ± 0.13**	**-0.15 ± 0.03**	**-0.06 ± 0.03**	**-0.15 ± 0.09**
			Peak Positive	**0.24 ± 0.11**	**0.47 ± 0.42**	**1.04 ± 0.16**	**0.33 ± 0.18**	**0.14 ± 0.05**	**0.25 ± 0.12**
Work [J/kg]	Device	Hip (Frontal)	Negative	-0.00 ± 0.00	-0.01 ± 0.00	**-0.00 ± 0.00**	**-0.02 ± 0.01**	-0.00 ± 0.00	-0.00 ± 0.00
			Positive	0.00 ± 0.00	0.00 ± 0.00	0.01 ± 0.01	0.01 ± 0.01	0.00 ± 0.00	0.00 ± 0.00
		Hip (Sagittal)	Negative	-0.04 ± 0.01	-0.03 ± 0.02	-0.10 ± 0.04	-0.11 ± 0.03	-0.07 ± 0.02	-0.07 ± 0.02
			Positive	0.05 ± 0.01	0.03 ± 0.02	**0.10 ± 0.03**	**0.24 ± 0.07**	0.06 ± 0.02	0.10 ± 0.04
	Intact	Hip (Frontal)	Negative	-0.01 ± 0.01	-0.01 ± 0.01	**-0.06 ± 0.01**	**-0.08 ± 0.04**	-0.04 ± 0.02	-0.03 ± 0.02
			Positive	0.02 ± 0.01	0.02 ± 0.01	**0.04 ± 0.01**	**0.03 ± 0.01**	0.01 ± 0.01	0.01 ± 0.00
		Hip (Sagittal)	Negative	**-0.03 ± 0.02**	**-0.07 ± 0.05**	-0.03 ± 0.03	-0.04 ± 0.03	**-0.15 ± 0.04**	**-0.09 ± 0.01**
			Positive	**0.53 ± 0.21**	**0.79 ± 0.28**	0.36 ± 0.11	0.41 ± 0.08	**1.20 ± 0.16**	**1.09 ± 0.13**
		Knee (Sagittal)	Negative	**-0.08 ± 0.06**	**-0.05 ± 0.04**	-0.01 ± 0.01	-0.00 ± 0.01	**-0.12 ± 0.06**	**-0.03 ± 0.03**
			Positive	**0.43 ± 0.19**	**0.68 ± 0.25**	0.97 ± 0.12	0.92 ± 0.19	**0.58 ± 0.08**	**1.34 ± 0.13**
		Ankle (Sagittal)	Negative	**-0.04 ± 0.02**	**-0.03 ± 0.02**	**-0.04 ± 0.02**	**-0.03 ± 0.01**	-0.01 ± 0.00	-0.01 ± 0.01
			Positive	0.05 ± 0.02	0.06 ± 0.04	**0.30 ± 0.04**	**0.11 ± 0.06**	**0.03 ± 0.01**	**0.07 ± 0.04**
	Total Works		Total Negative Work	-0.21	-0.20	-0.24	-0.28	**-0.39**	**-0.25**
			Total Positive Work	**1.08**	**1.59**	**1.79**	**1.73**	**1.89**	**2.61**
			Weighted Absolute Work	**5.38**	**7.81**	**8.83**	**8.59**	**9.43**	**12.78**

TABLE S-II
STAND-TO-SIT KINETICS

				Subject 1		Subject 2		Subject 3	
				Powered	Passive	Powered	Passive	Powered	Passive
GRF [N/kg]	Device Side		Peak Vertical	4.04 ± 0.43	3.82 ± 0.71	4.31 ± 0.04	4.65 ± 0.76	**4.94 ± 0.42**	**6.44 ± 2.80**
			Peak Propulsion	-0.25 ± 0.18	-0.10 ± 0.07	-0.40 ± 0.05	-0.12 ± 0.04	**-0.06 ± 0.02**	**-0.76 ± 0.82**
			Peak Braking	**1.45 ± 0.07**	**0.99 ± 0.11**	**1.28 ± 0.09**	**0.92 ± 0.17**	0.46 ± 0.15	0.55 ± 0.44
	Intact Side		Peak Vertical	7.36 ± 1.11	7.24 ± 2.93	**7.43 ± 1.50**	**9.66 ± 0.55**	**9.36 ± 1.22**	**10.51 ± 0.68**
			Peak Propulsion	-0.56 ± 0.22	-0.70 ± 0.38	-0.41 ± 0.11	-0.52 ± 0.14	-0.55 ± 0.19	-1.01 ± 0.48
			Peak Braking	**0.36 ± 0.17**	**0.89 ± 0.34**	**0.50 ± 0.12**	**1.20 ± 0.48**	**0.39 ± 0.16**	**1.01 ± 0.24**
Moment [Nm/kg]	Device	Hip (Frontal)	Peak Abduction	-0.04 ± 0.02	-0.05 ± 0.02	**-0.49 ± 0.05**	**-0.10 ± 0.04**	**-0.07 ± 0.03**	**-0.35 ± 0.17**
			Peak Adduction	**0.21 ± 0.06**	**0.40 ± 0.06**	0.09 ± 0.08	0.11 ± 0.04	**0.09 ± 0.03**	**0.52 ± 0.57**
		Hip (Sagittal)	Peak Extension	-0.18 ± 0.04	-0.22 ± 0.15	**-0.04 ± 0.02**	**-0.42 ± 0.06**	-0.30 ± 0.07	-0.22 ± 0.20
			Peak Flexion	**0.17 ± 0.04**	**0.40 ± 0.03**	**0.28 ± 0.05**	**0.20 ± 0.04**	**0.23 ± 0.05**	**0.41 ± 0.06**
	Intact	Hip (Frontal)	Peak Abduction	-0.38 ± 0.11	-0.34 ± 0.06	**-0.06 ± 0.04**	**-0.28 ± 0.27**	-0.29 ± 0.14	-0.25 ± 0.15
			Peak Adduction	0.18 ± 0.14	0.30 ± 0.28	**0.91 ± 0.23**	**0.74 ± 0.16**	**0.41 ± 0.13**	**0.56 ± 0.20**
		Hip (Sagittal)	Peak Extension	**-1.27 ± 0.23**	**-0.58 ± 0.58**	-0.65 ± 0.11	-0.65 ± 0.09	**-1.54 ± 0.15**	**-1.76 ± 0.22**
			Peak Flexion	**0.17 ± 0.16**	**0.43 ± 0.06**	**0.15 ± 0.09**	**0.21 ± 0.12**	**0.12 ± 0.13**	**0.29 ± 0.10**
		Knee (Frontal)	Peak Abduction	**-0.50 ± 0.13**	**-0.59 ± 0.24**	-0.19 ± 0.11	-0.24 ± 0.16	**-0.49 ± 0.12**	**-0.74 ± 0.13**
			Peak Adduction	0.05 ± 0.02	0.08 ± 0.06	**0.13 ± 0.07**	**0.25 ± 0.05**	**0.15 ± 0.09**	**0.33 ± 0.20**
		Knee (Sagittal)	Peak Extension	**-0.75 ± 0.20**	**-0.98 ± 0.51**	**-1.08 ± 0.10**	**-1.28 ± 0.20**	**-0.90 ± 0.10**	**-1.39 ± 0.29**
			Peak Flexion	0.20 ± 0.10	0.15 ± 0.08	0.09 ± 0.07	0.09 ± 0.06	0.27 ± 0.08	0.24 ± 0.17
		Ankle (Sagittal)	Peak Plantarflexion	**-0.86 ± 0.22**	**-0.40 ± 0.24**	**-0.69 ± 0.11**	**-0.83 ± 0.10**	**-0.60 ± 0.13**	**-1.00 ± 0.15**
			Peak Dorsiflexion	0.00 ± 0.00	0.02 ± 0.02	0.00 ± 0.00	0.00 ± 0.00	0.05 ± 0.07	0.08 ± 0.03
Power [W/kg]	Device	Hip (Frontal)	Peak Negative	**-0.05 ± 0.03**	**-0.22 ± 0.15**	-0.04 ± 0.03	-0.05 ± 0.03	**-0.04 ± 0.01**	**-0.18 ± 0.11**
			Peak Positive	**0.05 ± 0.03**	**0.28 ± 0.16**	**0.19 ± 0.06**	**0.07 ± 0.04**	**0.05 ± 0.03**	**0.20 ± 0.12**
		Hip (Sagittal)	Peak Negative	**-0.17 ± 0.08**	**-0.35 ± 0.14**	-0.18 ± 0.06	-0.33 ± 0.08	**-0.17 ± 0.05**	**-0.41 ± 0.26**
			Peak Positive	**0.30 ± 0.11**	**0.44 ± 0.10**	0.25 ± 0.05	0.27 ± 0.08	0.42 ± 0.14	0.43 ± 0.22
	Intact	Hip (Frontal)	Peak Negative	**-0.10 ± 0.04**	**-0.23 ± 0.11**	**-0.40 ± 0.20**	**-0.29 ± 0.13**	-0.30 ± 0.17	-0.37 ± 0.16
			Peak Positive	0.18 ± 0.10	0.17 ± 0.10	**0.16 ± 0.11**	**0.42 ± 0.16**	0.17 ± 0.07	0.23 ± 0.13
		Hip (Sagittal)	Peak Negative	**-1.98 ± 0.51**	**-1.67 ± 0.45**	-0.67 ± 0.08	-0.77 ± 0.15	**-1.73 ± 0.33**	**-1.95 ± 0.51**
			Peak Positive	**0.31 ± 0.16**	**0.48 ± 0.22**	0.10 ± 0.02	0.10 ± 0.06	0.47 ± 0.17	0.53 ± 0.25
		Knee (Sagittal)	Peak Negative	**-1.26 ± 0.37**	**-0.92 ± 0.44**	**-1.87 ± 0.33**	**-2.14 ± 0.42**	-1.93 ± 0.26	-1.79 ± 0.46
			Peak Positive	**0.32 ± 0.21**	**0.20 ± 0.11**	0.08 ± 0.06	0.11 ± 0.09	**0.55 ± 0.27**	**0.22 ± 0.12**
		Ankle (Sagittal)	Peak Negative	**-0.58 ± 0.27**	**-0.13 ± 0.05**	-0.53 ± 0.17	-0.53 ± 0.15	**-0.33 ± 0.12**	**-0.59 ± 0.20**
			Peak Positive	**0.50 ± 0.33**	**0.20 ± 0.15**	**0.24 ± 0.12**	**0.40 ± 0.18**	0.37 ± 0.19	0.38 ± 0.23
Work [J/kg]	Device	Hip (Frontal)	Negative	**-0.01 ± 0.00**	**-0.04 ± 0.03**	-0.00 ± 0.00	-0.01 ± 0.00	**-0.01 ± 0.00**	**-0.03 ± 0.02**
			Positive	**0.01 ± 0.01**	**0.08 ± 0.03**	**0.07 ± 0.02**	**0.01 ± 0.02**	**0.01 ± 0.00**	**0.03 ± 0.02**
		Hip (Sagittal)	Negative	-0.03 ± 0.02	-0.05 ± 0.02	-0.32 ± 0.04	-0.35 ± 0.04	-0.05 ± 0.03	-0.07 ± 0.06
			Positive	**0.07 ± 0.02**	**0.22 ± 0.07**	0.09 ± 0.02	0.12 ± 0.03	**0.08 ± 0.03**	**0.17 ± 0.08**
	Intact	Hip (Frontal)	Negative	**-0.02 ± 0.01**	**-0.04 ± 0.02**	-0.07 ± 0.03	-0.06 ± 0.02	**-0.04 ± 0.03**	**-0.09 ± 0.06**
			Positive	**0.03 ± 0.01**	**0.05 ± 0.03**	**0.02 ± 0.01**	**0.09 ± 0.04**	0.04 ± 0.02	0.03 ± 0.01
		Hip (Sagittal)	Negative	**-0.65 ± 0.10**	**-0.81 ± 0.20**	-0.32 ± 0.04	-0.35 ± 0.04	-0.73 ± 0.08	-0.77 ± 0.15
			Positive	**0.04 ± 0.02**	**0.08 ± 0.04**	0.01 ± 0.01	0.04 ± 0.01	0.06 ± 0.02	0.06 ± 0.02
		Knee (Sagittal)	Negative	-0.34 ± 0.14	-0.39 ± 0.25	-0.80 ± 0.09	-0.84 ± 0.17	**-0.43 ± 0.05**	**-0.85 ± 0.29**
			Positive	**0.06 ± 0.05**	**0.04 ± 0.03**	0.01 ± 0.01	0.01 ± 0.01	**0.09 ± 0.06**	**0.02 ± 0.01**
		Ankle (Sagittal)	Negative	**-0.17 ± 0.05**	**-0.04 ± 0.03**	-0.17 ± 0.04	-0.16 ± 0.04	**-0.10 ± 0.04**	**-0.23 ± 0.08**
			Positive	**0.08 ± 0.04**	**0.02 ± 0.01**	0.03 ± 0.02	0.04 ± 0.02	0.05 ± 0.03	0.06 ± 0.04
	Total Works		Total Negative Work	**-1.22**	**-1.38**	**-1.38**	**-1.49**	**-1.36**	**-2.05**
			Total Positive Work	**0.29**	**0.49**	**0.23**	**0.30**	0.33	0.37
			Weighted Absolute Work	**2.60**	**3.71**	**2.49**	**2.92**	**2.95**	**3.83**

TABLE S-III
LEVEL WALK KINETICS

				Subject 1		Subject 2		Subject 3	
				Powered	Passive	Powered	Passive	Powered	Passive
GRF [N/kg]	Device Side		Peak Vertical	**10.39 ± 0.60**	**13.31 ± 0.89**	**10.64 ± 0.68**	**11.24 ± 0.56**	**9.12 ± 0.38**	**10.91 ± 0.54**
			Peak Propulsion	**-2.13 ± 0.39**	**-1.43 ± 0.22**	**-1.87 ± 0.18**	**-1.45 ± 0.18**	**-0.81 ± 0.30**	**-1.09 ± 0.11**
			Peak Braking	**1.69 ± 0.31**	**2.22 ± 0.44**	1.54 ± 0.28	1.63 ± 0.22	**0.20 ± 0.14**	**0.96 ± 0.86**
	Intact Side		Peak Vertical	**12.44 ± 0.86**	**13.93 ± 1.04**	**12.41 ± 0.32**	**11.77 ± 0.41**	11.82 ± 0.62	12.02 ± 0.70
			Peak Propulsion	-2.94 ± 0.17	-2.97 ± 0.07	-2.94 ± 0.17	-2.76 ± 0.21	**-1.08 ± 0.17**	**-1.89 ± 0.69**
			Peak Braking	2.63 ± 0.25	2.62 ± 0.32	**2.94 ± 0.14**	**2.45 ± 0.27**	**0.10 ± 0.07**	**0.96 ± 0.98**
Moment [Nm/kg]	Device	Hip (Frontal)	Peak Abduction	-0.10 ± 0.06	-0.14 ± 0.03	**-0.23 ± 0.23**	**-0.04 ± 0.02**	-0.07 ± 0.04	-0.11 ± 0.11
			Peak Adduction	**0.61 ± 0.10**	**0.92 ± 0.09**	**1.02 ± 0.10**	**0.88 ± 0.06**	0.47 ± 0.07	0.42 ± 0.08
		Hip (Sagittal)	Peak Extension	**-0.71 ± 0.26**	**-0.38 ± 0.13**	**-0.82 ± 0.19**	**-0.32 ± 0.14**	**-0.66 ± 0.23**	**-0.21 ± 0.06**
			Peak Flexion	**0.84 ± 0.12**	**1.46 ± 0.16**	**0.59 ± 0.11**	**1.30 ± 0.10**	**0.72 ± 0.15**	**1.26 ± 0.07**
	Intact	Hip (Frontal)	Peak Abduction	-0.32 ± 0.08	-0.30 ± 0.06	**-0.33 ± 0.07**	**-0.22 ± 0.04**	**-0.36 ± 0.06**	**-0.29 ± 0.05**
			Peak Adduction	**0.76 ± 0.09**	**1.04 ± 0.13**	**1.48 ± 0.05**	**1.14 ± 0.08**	**1.05 ± 0.18**	**0.66 ± 0.08**
		Hip (Sagittal)	Peak Extension	**-1.06 ± 0.23**	**-1.58 ± 0.42**	-0.56 ± 0.14	-0.48 ± 0.17	**-0.96 ± 0.17**	**-0.70 ± 0.15**
			Peak Flexion	**0.65 ± 0.12**	**0.90 ± 0.15**	**0.74 ± 0.10**	**0.84 ± 0.09**	0.91 ± 0.29	0.91 ± 0.11
		Knee (Frontal)	Peak Abduction	**-0.14 ± 0.04**	**-0.20 ± 0.12**	**-0.26 ± 0.04**	**-0.18 ± 0.06**	**-0.18 ± 0.07**	**-0.37 ± 0.18**
			Peak Adduction	**0.31 ± 0.16**	**0.41 ± 0.12**	**0.30 ± 0.05**	**0.24 ± 0.03**	**0.47 ± 0.07**	**0.41 ± 0.09**
		Knee (Sagittal)	Peak Extension	-1.29 ± 0.26	-1.38 ± 0.17	-1.16 ± 0.14	-1.09 ± 0.10	-1.5 ± 0.25	-1.55 ± 0.21
			Peak Flexion	**0.43 ± 0.06**	**0.63 ± 0.24**	0.35 ± 0.06	0.32 ± 0.06	**0.51 ± 0.10**	**0.41 ± 0.05**
		Ankle (Sagittal)	Peak Plantarflexion	**-1.77 ± 0.14**	**-1.67 ± 0.09**	**-1.89 ± 0.07**	**-1.62 ± 0.08**	-1.58 ± 0.13	-1.54 ± 0.08
			Peak Dorsiflexion	**0.39 ± 0.12**	**0.54 ± 0.38**	**0.32 ± 0.06**	**0.26 ± 0.12**	**0.44 ± 0.09**	**0.31 ± 0.07**
Power [W/kg]	Device	Hip (Frontal)	Peak Negative	**-0.47 ± 0.13**	**-0.67 ± 0.11**	**-0.91 ± 0.21**	**-1.04 ± 0.11**	-0.14 ± 0.04	-0.17 ± 0.05
			Peak Positive	**0.19 ± 0.10**	**0.46 ± 0.28**	0.46 ± 0.14	0.47 ± 0.13	0.11 ± 0.06	0.06 ± 0.02
		Hip (Sagittal)	Peak Negative	**-1.14 ± 0.20**	**-1.75 ± 0.19**	**-0.98 ± 0.22**	**-1.71 ± 0.16**	**-1.19 ± 0.25**	**-1.32 ± 0.14**
			Peak Positive	**1.00 ± 0.18**	**1.95 ± 0.39**	**1.45 ± 0.26**	**0.65 ± 0.10**	**0.60 ± 0.16**	**0.91 ± 0.09**
	Intact	Hip (Frontal)	Peak Negative	**-0.63 ± 0.17**	**-0.83 ± 0.26**	-1.15 ± 0.19	-1.17 ± 0.21	**-0.45 ± 0.12**	**-0.37 ± 0.07**
			Peak Positive	0.53 ± 0.28	0.57 ± 0.30	**0.96 ± 0.31**	**0.40 ± 0.14**	0.22 ± 0.15	0.17 ± 0.10
		Hip (Sagittal)	Peak Negative	**-0.76 ± 0.20**	**-1.34 ± 0.23**	**-0.49 ± 0.12**	**-0.93 ± 0.15**	-1.21 ± 0.42	-1.18 ± 0.25
			Peak Positive	**1.13 ± 0.19**	**1.64 ± 0.28**	0.81 ± 0.13	0.75 ± 0.13	**1.49 ± 0.31**	**1.07 ± 0.22**
		Knee (Sagittal)	Peak Negative	-2.21 ± 0.46	-1.99 ± 0.57	**-2.32 ± 0.37**	**-1.51 ± 0.37**	**-3.08 ± -0.71**	**-2.08 ± 0.45**
			Peak Positive	1.33 ± 0.30	1.44 ± 0.23	1.12 ± 0.18	0.98 ± 0.18	1.54 ± 0.37	1.40 ± 0.38
		Ankle (Sagittal)	Peak Negative	**-1.18 ± 0.26**	**-1.35 ± 0.38**	**-1.86 ± 0.34**	**-1.26 ± 0.09**	**-0.66 ± 0.28**	**-0.29 ± 0.11**
			Peak Positive	**4.78 ± 0.64**	**4.24 ± 0.40**	**3.98 ± 0.35**	**3.09 ± 0.39**	**3.77 ± 0.70**	**3.17 ± 0.50**
Work [J/kg]	Device	Hip (Frontal)	Negative	-0.09 ± 0.02	-0.09 ± 0.02	**-0.15 ± 0.04**	**-0.22 ± 0.02**	-0.03 ± 0.01	-0.05 ± 0.01
			Positive	**0.02 ± 0.01**	**0.07 ± 0.03**	**0.08 ± 0.03**	**0.05 ± 0.01**	**0.02 ± 0.01**	**0.01 ± 0.00**
		Hip (Sagittal)	Negative	**-0.18 ± 0.05**	**-0.27 ± 0.07**	**-0.21 ± 0.06**	**-0.42 ± 0.08**	**-0.28 ± 0.05**	**-0.31 ± 0.05**
			Positive	0.26 ± 0.09	0.26 ± 0.06	**0.29 ± 0.06**	**0.07 ± 0.02**	0.12 ± 0.06	0.12 ± 0.02
	Intact	Hip (Frontal)	Negative	**-0.14 ± 0.03**	**-0.16 ± 0.05**	-0.32 ± 0.04	-0.30 ± 0.03	**-0.17 ± 0.05**	**-0.11 ± 0.02**
			Positive	**0.04 ± 0.02**	**0.06 ± 0.02**	**0.12 ± 0.03**	**0.06 ± 0.02**	0.02 ± 0.01	0.01 ± 0.01
		Hip (Sagittal)	Negative	**-0.19 ± 0.06**	**-0.25 ± 0.01**	**-0.13 ± 0.04**	**-0.32 ± 0.06**	**-0.28 ± 0.11**	**-0.36 ± 0.07**
			Positive	**0.35 ± 0.07**	**0.39 ± 0.07**	**0.20 ± 0.02**	**0.15 ± 0.04**	**0.35 ± 0.04**	**0.20 ± 0.02**
		Knee (Sagittal)	Negative	**-0.47 ± 0.08**	**-0.41 ± 0.07**	**-0.38 ± 0.05**	**-0.32 ± 0.07**	**-0.62 ± 0.10**	**-0.44 ± 0.06**
			Positive	0.20 ± 0.05	0.19 ± 0.05	0.16 ± 0.03	0.17 ± 0.03	**0.31 ± 0.07**	**0.22 ± 0.05**
		Ankle (Sagittal)	Negative	**-0.21 ± 0.05**	**-0.17 ± 0.06**	**-0.33 ± 0.05**	**-0.25 ± 0.03**	**-0.12 ± 0.05**	**-0.05 ± 0.02**
			Positive	**0.58 ± 0.09**	**0.47 ± 0.03**	**0.57 ± 0.04**	**0.44 ± 0.05**	0.47 ± 0.08	0.47 ± 0.06
	Total Works		Total Negative Work	-1.30	-1.35	**-1.52**	**-1.83**	**-1.49**	**-1.31**
			Total Positive Work	1.45	1.44	**1.40**	**0.95**	**1.30**	**1.04**
			Weighted Absolute Work	8.24	8.26	**8.24**	**6.37**	**7.72**	**6.33**

TABLE S-IV
STAIR DESCENT KINETICS

				Subject 1		Subject 2		Subject 3	
				Powered	Passive	Powered	Passive	Powered	Passive
GRF [N/kg]	Device Side		Peak Vertical	**6.85 ± 0.87**	**9.94 ± 1.16**	**8.44 ± 0.46**	**9.97 ± 0.85**	10.11 ± 1.11	9.87 ± 0.81
			Peak Propulsion	**-1.22 ± 0.24**	**-2.46 ± 0.28**	**-0.71 ± 0.07**	**-0.09 ± 0.05**	**-0.52 ± 0.10**	**-1.58 ± 0.61**
			Peak Braking	0.47 ± 0.14	0.52 ± 0.41	**1.17 ± 0.10**	**0.46 ± 0.12**	0.09 ± 0.03	0.20 ± 0.12
	Intact Side		Peak Vertical	**12.06 ± 0.85**	**14.30 ± 0.86**	11.12 ± 1.85	11.03 ± 0.87	**11.71 ± 1.99**	**14.08 ± 1.10**
			Peak Propulsion	**-1.89 ± 0.31**	**-1.64 ± 0.32**	**-1.45 ± 0.37**	**-2.25 ± 0.38**	**-0.36 ± 0.11**	**-1.01 ± 0.73**
			Peak Braking	**1.22 ± 0.21**	**1.90 ± 0.35**	**0.88 ± 0.49**	**0.48 ± 0.36**	**0.57 ± 0.11**	**0.83 ± 0.41**
Moment [Nm/kg]	Device	Hip (Frontal)	Peak Abduction	**-0.21 ± 0.03**	**-0.26 ± 0.11**	-0.05 ± 0.03	-0.02 ± 0.02	-0.30 ± 0.05	-0.30 ± 0.13
			Peak Adduction	**0.17 ± 0.03**	**0.30 ± 0.12**	**0.75 ± 0.62**	**0.57 ± 0.06**	0.22 ± 0.06	0.19 ± 0.07
		Hip (Sagittal)	Peak Extension	**-0.22 ± 0.10**	**-0.46 ± 0.19**	-0.23 ± 0.08	-0.25 ± 0.12	**-0.24 ± 0.09**	**-0.15 ± 0.05**
			Peak Flexion	**0.57 ± 0.08**	**1.95 ± 0.20**	0.44 ± 0.11	0.51 ± 0.14	**0.99 ± 0.26**	**1.75 ± 0.32**
	Intact	Hip (Frontal)	Peak Abduction	**-0.17 ± 0.05**	**-0.28 ± 0.07**	-0.25 ± 0.07	-0.31 ± 0.07	-0.31 ± 0.11	-0.29 ± 0.11
			Peak Adduction	**0.75 ± 0.09**	**0.98 ± 0.15**	**1.12 ± 0.17**	**0.61 ± 0.12**	**0.83 ± 0.16**	**0.67 ± 0.21**
		Hip (Sagittal)	Peak Extension	**-0.38 ± 0.10**	**-0.54 ± 0.09**	**-0.19 ± 0.05**	**-0.34 ± 0.04**	**-0.19 ± 0.06**	**-0.43 ± 0.07**
			Peak Flexion	**0.32 ± 0.05**	**0.59 ± 0.22**	**0.87 ± 0.53**	**1.36 ± 0.37**	**0.97 ± 0.27**	**1.16 ± 0.38**
		Knee (Frontal)	Peak Abduction	**-0.27 ± 0.08**	**-0.48 ± 0.13**	**-0.39 ± 0.14**	**-0.63 ± 0.16**	**-0.44 ± 0.13**	**-0.56 ± 0.17**
			Peak Adduction	0.17 ± 0.08	0.17 ± 0.04	**0.18 ± 0.06**	**0.11 ± 0.03**	**0.25 ± 0.09**	**0.14 ± 0.03**
		Knee (Sagittal)	Peak Extension	**-1.80 ± 0.19**	**-1.93 ± 0.27**	**-1.57 ± 0.08**	**-1.85 ± 0.24**	**-1.49 ± 0.22**	**-1.77 ± 0.29**
			Peak Flexion	**0.24 ± 0.04**	**0.41 ± 0.05**	0.27 ± 0.06	0.31 ± 0.03	**0.23 ± 0.04**	**0.33 ± 0.04**
		Ankle (Sagittal)	Peak Plantarflexion	**-1.63 ± 0.12**	**-1.28 ± 0.25**	**-0.84 ± 0.23**	**-0.70 ± 0.17**	**-0.81 ± 0.13**	**-1.11 ± 0.33**
			Peak Dorsiflexion	**0.28 ± 0.18**	**0.11 ± 0.02**	0.07 ± 0.01	0.09 ± 0.01	0.08 ± 0.02	0.11 ± 0.02
Power [W/kg]	Device	Hip (Frontal)	Peak Negative	**-0.09 ± 0.03**	**-0.15 ± 0.07**	-0.24 ± 0.06	-0.20 ± 0.07	-0.08 ± 0.02	-0.12 ± 0.05
			Peak Positive	0.15 ± 0.04	0.10 ± 0.06	**0.39 ± 0.09**	**0.08 ± 0.03**	0.09 ± 0.04	0.13 ± 0.05
		Hip (Sagittal)	Peak Negative	-0.56 ± 0.12	-0.60 ± 0.23	-0.32 ± 0.13	-0.16 ± 0.05	**-1.46 ± 0.36**	**-0.74 ± 0.22**
			Peak Positive	**0.67 ± 0.14**	**2.01 ± 0.53**	0.35 ± 0.09	0.33 ± 0.13	**0.75 ± 0.16**	**1.66 ± 0.51**
	Intact	Hip (Frontal)	Peak Negative	**-0.37 ± 0.09**	**-0.45 ± 0.18**	**-0.67 ± 0.13**	**-0.27 ± 0.14**	**-0.45 ± 0.13**	**0.24 ± 0.14**
			Peak Positive	**0.39 ± 0.11**	**0.57 ± 0.21**	**0.74 ± 0.12**	**0.53 ± 0.15**	0.34 ± 0.11	0.28 ± 0.07
		Hip (Sagittal)	Peak Negative	**-0.32 ± 0.14**	**-0.53 ± 0.22**	**-0.74 ± 0.63**	**-1.76 ± 0.67**	**-0.29 ± 0.20**	**-0.69 ± 0.33**
			Peak Positive	**0.50 ± 0.12**	**1.14 ± 0.25**	**0.68 ± 0.12**	**1.46 ± 0.42**	0.87 ± 0.30	0.87 ± 0.20
		Knee (Sagittal)	Peak Negative	**-3.13 ± 0.34**	**-3.76 ± 0.62**	**-2.62 ± 0.34**	**-3.95 ± 0.59**	**-3.50 ± 0.62**	**-3.12 ± 0.49**
			Peak Positive	**1.55 ± 0.42**	**0.40 ± 0.15**	**0.80 ± 0.38**	**2.62 ± 0.57**	**1.07 ± 0.48**	**0.85 ± 0.36**
		Ankle (Sagittal)	Peak Negative	**-1.68 ± 0.41**	**-3.07 ± 0.67**	**-2.17 ± 0.22**	**-1.63 ± 0.33**	**-1.57 ± 0.49**	**-2.62 ± 0.60**
			Peak Positive	2.85 ± 0.45	2.79 ± 0.64	**1.28 ± 0.67**	**0.66 ± 0.44**	**0.78 ± 0.35**	**1.85 ± 1.19**
Work [J/kg]	Device	Hip (Frontal)	Negative	**-0.02 ± 0.01**	**-0.03 ± 0.01**	**-0.05 ± 0.01**	**-0.03 ± 0.01**	-0.02 ± 0.01	-0.03 ± 0.01
			Positive	**0.03 ± 0.01**	**0.01 ± 0.01**	**0.09 ± 0.02**	**0.02 ± 0.01**	0.02 ± 0.01	0.02 ± 0.01
		Hip (Sagittal)	Negative	**-0.12 ± 0.02**	**-0.08 ± 0.03**	**-0.08 ± 0.04**	**-0.02 ± 0.01**	**-0.16 ± 0.06**	**-0.12 ± 0.05**
			Positive	**0.16 ± 0.02**	**0.50 ± 0.12**	0.08 ± 0.01	0.06 ± 0.02	**0.18 ± 0.03**	**0.41 ± 0.10**
	Intact	Hip (Frontal)	Negative	**-0.09 ± 0.03**	**-0.07 ± 0.03**	**-0.13 ± 0.02**	**-0.04 ± 0.02**	**-0.07 ± 0.02**	**-0.03 ± 0.02**
			Positive	0.12 ± 0.03	0.11 ± 0.03	**0.23 ± 0.02**	**0.12 ± 0.03**	**0.10 ± 0.03**	**0.07 ± 0.01**
		Hip (Sagittal)	Negative	**-0.04 ± 0.02**	**-0.08 ± 0.03**	**-0.12 ± 0.10**	**-0.21 ± 0.07**	**-0.04 ± 0.03**	**-0.12 ± 0.06**
			Positive	**0.17 ± 0.04**	**0.26 ± 0.05**	**0.26 ± 0.06**	**0.49 ± 0.11**	0.19 ± 0.05	0.24 ± 0.06
		Knee (Sagittal)	Negative	**-1.47 ± 0.18**	**-1.32 ± 0.13**	**-1.01 ± 0.06**	**-1.41 ± 0.11**	-1.17 ± 0.10	-1.25 ± 0.11
			Positive	**0.23 ± 0.09**	**0.04 ± 0.02**	**0.10 ± 0.03**	**0.32 ± 0.07**	**0.12 ± 0.05**	**0.10 ± 0.04**
		Ankle (Sagittal)	Negative	**-0.54 ± 0.10**	**-0.51 ± 0.10**	**-0.62 ± 0.06**	**-0.39 ± 0.10**	**-0.40 ± 0.12**	**-0.51 ± 0.11**
			Positive	**0.33 ± 0.06**	**0.29 ± 0.07**	**0.17 ± 0.09**	**0.09 ± 0.06**	**0.10 ± 0.05**	**0.20 ± 0.14**
	Total Works		Total Negative Work	**-2.27**	**-2.08**	**-2.01**	**-2.10**	**-1.87**	**-2.06**
			Total Positive Work	**1.03**	**1.21**	**0.93**	**1.10**	**0.72**	**1.03**
			Weighted Absolute Work	**7.21**	**7.88**	**6.49**	**7.36**	**5.31**	**7.00**

TABLE S-V
STAIR ASCENT KINETICS

				Subject 1		Subject 2		Subject 3	
				Powered	Passive	Powered	Passive	Powered	Passive
GRF [N/kg]	Device Side		Peak Vertical	**9.36 ± 0.57**	**8.11 ± 0.91**	**8.51 ± 1.03**	**11.30 ± 0.87**	**9.68 ± 0.48**	**8.63 ± 1.01**
			Peak Propulsion	**-1.07 ± 0.12**	**-0.68 ± 0.14**	**-0.36 ± 0.0**	**-0.80 ± 0.16**	-0.12 ± 0.04	-0.17 ± 0.17
			Peak Braking	**0.50 ± 0.09**	**0.17 ± 0.10**	**0.63 ± 0.10**	**0.00 ± 0.00**	0.84 ± 0.11	0.99 ± 0.16
	Intact Side		Peak Vertical	**10.29 ± 0.73**	**11.69 ± 0.95**	**10.66 ± 0.35**	**9.08 ± 1.06**	**10.99 ± 0.63**	**12.00 ± 1.07**
			Peak Propulsion	**-1.11 ± 0.14**	**-1.79 ± 0.23**	**-0.84 ± 0.07**	**-0.53 ± 0.21**	**-0.55 ± 0.11**	**-1.18 ± 0.27**
			Peak Braking	1.23 ± 0.39	1.19 ± 0.43	1.02 ± 0.39	1.17 ± 0.38	**0.18 ± 0.07**	**0.65 ± 0.77**
Moment [Nm/kg]	Device	Hip (Frontal)	Peak Abduction	**-0.16 ± 0.07**	**-0.29 ± 0.07**	-0.05 ± 0.03	-0.09 ± 0.03	**-0.09 ± 0.06**	**0.31 ± 0.17**
			Peak Adduction	0.51 ± 0.09	0.58 ± 0.16	**0.56 ± 0.05**	**1.22 ± 0.14**	**0.17 ± 0.02**	**0.48 ± 0.12**
		Hip (Sagittal)	Peak Extension	-0.60 ± 0.07	-0.65 ± 0.22	**-0.42 ± 0.07**	**-0.22 ± 0.09**	-0.42 ± 0.13	-0.55 ± 0.18
			Peak Flexion	0.21 ± 0.05	0.25 ± 0.07	**0.41 ± 0.07**	**0.62 ± 0.08**	**0.43 ± 0.06**	**0.23 ± 0.11**
	Intact	Hip (Frontal)	Peak Abduction	**-0.21 ± 0.05**	**-0.17 ± 0.04**	**-0.22 ± 0.02**	**-0.14 ± 0.04**	**-0.37 ± 0.08**	**-0.24 ± 0.07**
			Peak Adduction	**0.52 ± 0.21**	**0.88 ± 0.13**	**1.24 ± 0.09**	**0.81 ± 0.13**	**0.66 ± 0.07**	**0.49 ± 0.16**
		Hip (Sagittal)	Peak Extension	**-1.42 ± 0.31**	**-1.66 ± 0.38**	**-0.85 ± 0.24**	**-0.51 ± 0.26**	**-1.57 ± 0.33**	**-1.25 ± 0.26**
			Peak Flexion	**0.20 ± 0.06**	**0.25 ± 0.06**	**0.25 ± 0.10**	**0.45 ± 0.10**	**0.24 ± 0.05**	**0.19 ± 0.08**
		Knee (Frontal)	Peak Abduction	**-0.22 ± 0.15**	**-0.32 ± 0.16**	**-0.19 ± 0.09**	**-0.11 ± 0.08**	**-0.24 ± 0.07**	**-0.32 ± 0.17**
			Peak Adduction	**0.33 ± 0.13**	**0.80 ± 0.09**	**0.33 ± 0.02**	**0.50 ± 0.09**	0.37 ± 0.05	0.37 ± 0.10
		Knee (Sagittal)	Peak Extension	-1.15 ± 0.30	-1.16 ± 0.40	**-1.37 ± 0.30**	**-1.08 ± 0.31**	**-0.63 ± 0.29**	**-1.33 ± 0.39**
			Peak Flexion	**0.71 ± 0.12**	**0.87 ± 0.13**	**0.35 ± 0.06**	**0.23 ± 0.11**	0.45 ± 0.08	0.48 ± 0.14
		Ankle (Sagittal)	Peak Plantarflexion	-1.27 ± 0.13	-1.23 ± 0.11	**-1.15 ± 0.07**	**-0.97 ± 0.15**	**-1.37 ± 0.09**	**-1.43 ± 0.12**
			Peak Dorsiflexion	**0.06 ± 0.01**	**0.10 ± 0.04**	**0.06 ± 0.01**	**0.10 ± 0.02**	**0.08 ± 0.01**	**0.05 ± 0.01**
Power [W/kg]	Device	Hip (Frontal)	Peak Negative	**-0.49 ± 0.12**	**-0.75 ± 0.25**	**-0.54 ± 0.11**	**1.37 ± 0.41**	**-0.06 ± 0.02**	**-0.45 ± 0.17**
			Peak Positive	**0.54 ± 0.12**	**0.08 ± 0.05**	**0.21 ± 0.09**	**0.09 ± 0.02**	0.19 ± 0.03	0.23 ± 0.18
		Hip (Sagittal)	Peak Negative	**-0.74 ± 0.18**	**-0.24 ± 0.17**	**-0.48 ± 0.15**	**-0.16 ± 0.10**	**-0.43 ± 0.12**	**-0.10 ± 0.06**
			Peak Positive	1.31 ± 0.17	1.42 ± 0.54	**0.90 ± 0.20**	**0.26 ± 0.15**	**0.60 ± 0.14**	**1.18 ± 0.40**
	Intact	Hip (Frontal)	Peak Negative	**-0.53 ± 0.24**	**-0.83 ± 0.29**	**-1.90 ± 0.32**	**-0.70 ± 0.23**	-0.70 ± 0.20	-0.65 ± 0.23
			Peak Positive	**0.19 ± 0.07**	**0.27 ± 0.11**	0.38 ± 0.13	0.34 ± 0.10	0.17 ± 0.06	0.16 ± 0.05
		Hip (Sagittal)	Peak Negative	**-0.22 ± 0.12**	**-0.38 ± 0.17**	-0.35 ± 0.10	-0.37 ± 0.11	**-0.33 ± 0.11**	**-0.19 ± 0.10**
			Peak Positive	**1.96 ± 0.47**	**2.47 ± 0.51**	**1.35 ± 0.50**	**0.87 ± 0.26**	**2.47 ± 0.60**	**1.61 ± 0.40**
		Knee (Sagittal)	Peak Negative	**-0.80 ± 0.14**	**-1.01 ± 0.28**	**-0.88 ± 0.20**	**-0.21 ± 0.05**	**-1.19 ± 0.25**	**-0.60 ± 0.19**
			Peak Positive	**1.96 ± 0.65**	**2.16 ± 0.57**	**3.63 ± 0.72**	**2.25 ± 0.61**	**1.70 ± 0.37**	**2.29 ± 0.87**
		Ankle (Sagittal)	Peak Negative	**-0.68 ± 0.25**	**-0.96 ± 0.13**	**-1.09 ± 0.35**	**-1.81 ± 0.35**	**-0.99 ± 0.24**	**-1.11 ± 0.27**
			Peak Positive	2.06 ± 0.36	1.95 ± 0.33	**2.45 ± 0.41**	**1.99 ± 0.46**	**2.59 ± 0.57**	**3.05 ± 0.42**
Work [J/kg]	Device	Hip (Frontal)	Negative	-0.15 ± 0.03	-0.17 ± 0.05	**-0.12 ± 0.02**	**-0.16 ± 0.03**	**-0.02 ± 0.01**	**-0.12 ± 0.05**
			Positive	**0.07 ± 0.01**	**0.01 ± 0.01**	**0.04 ± 0.01**	**0.02 ± 0.00**	**0.04 ± 0.01**	**0.07 ± 0.07**
		Hip (Sagittal)	Negative	**-0.11 ± 0.02**	**-0.03 ± 0.03**	**-0.14 ± 0.05**	**-0.04 ± 0.02**	**-0.09 ± 0.03**	**-0.01 ± 0.01**
			Positive	0.36 ± 0.05	0.35 ± 0.11	**0.18 ± 0.04**	**0.04 ± 0.02**	**0.14 ± 0.04**	**0.32 ± 0.09**
	Intact	Hip (Frontal)	Negative	**-0.14 ± 0.06**	**-0.19 ± 0.05**	**-0.45 ± 0.05**	**-0.18 ± 0.05**	**-0.22 ± 0.04**	**-0.17 ± 0.06**
			Positive	**0.05 ± 0.02**	**0.03 ± 0.01**	0.06 ± 0.03	0.05 ± 0.02	0.03 ± 0.01	0.02 ± 0.01
		Hip (Sagittal)	Negative	**-0.03 ± 0.01**	**-0.04 ± 0.02**	**-0.05 ± 0.02**	**-0.07 ± 0.02**	**-0.04 ± 0.01**	**-0.02 ± 0.01**
			Positive	**0.93 ± 0.27**	**1.07 ± 0.19**	**0.43 ± 0.12**	**0.25 ± 0.09**	**0.85 ± 0.16**	**0.70 ± 0.11**
		Knee (Sagittal)	Negative	**-0.25 ± 0.09**	**-0.29 ± 0.07**	**-0.12 ± 0.04**	**-0.03 ± 0.01**	**-0.27 ± 0.06**	**-0.16 ± 0.06**
			Positive	**0.48 ± 0.16**	**0.55 ± 0.19**	**0.70 ± 0.13**	**0.60 ± 0.14**	**0.35 ± 0.12**	**0.50 ± 0.18**
		Ankle (Sagittal)	Negative	-0.14 ± 0.06	-0.13 ± 0.04	**-0.17 ± 0.07**	**-0.30 ± 0.05**	**-0.16 ± 0.04**	**-0.14 ± 0.04**
			Positive	**0.40 ± 0.07**	**0.63 ± 0.07**	**0.73 ± 0.06**	**0.43 ± 0.08**	**0.79 ± 0.08**	**0.96 ± 0.10**
	Total Works		Total Negative Work	-0.80	-0.85	**-1.06**	**-0.79**	**-0.79**	**-0.62**
			Total Positive Work	**2.30**	**2.64**	**2.12**	**1.39**	**2.21**	**2.57**
			Weighted Absolute Work	**11.83**	**13.53**	**11.25**	**7.44**	**11.41**	**12.98**

IV. Symmetry Analysis

Degree of asymmetry (DOA) between the intact-side and the device-side hips served as a secondary metric using

$$DOA = \frac{i-d}{i+d}$$

where i is intact side and d is device side [5]. $DOA = 0$ indicates perfect symmetry, negative shows bias towards device side, and positive shows bias towards intact side. Symmetry was only analyzed for the hips (frontal and sagittal planes) — to show the symmetry between *intact* joints on both sides — and the ground reaction forces (GRF). The symmetry results for each activity can be found in Tables S-VI through S-X.

TABLE S-VI
Sit-To-Stand Symmetry

		Subject 1		Subject 2		Subject 3	
		Powered	Passive	Powered	Passive	Powered	Passive
GRF	Vertical	0.38	0.68	0.26	0.40	0.42	0.70
	Propulsion	0.43	0.85	0.70	0.22	0.81	0.83
	Braking	-0.16	0.71	0.08	0.94	-0.16	0.85
Moment	Peak Abduction	0.83	-0.81	0.23	0.30	0.55	0.13
	Peak Adduction	0.26	-0.39	0.45	0.44	0.71	0.60
	Peak Extension	0.67	0.77	0.08	-0.02	0.74	0.63
	Peak Flexion	0.02	0.04	0.24	0.47	-0.09	0.42
Power	Peak Abduction	0.42	-0.12	0.79	0.34	0.79	0.70
	Peak Adduction	0.68	0.68	0.76	0.39	0.33	0.38
	Peak Extension	0.14	0.44	-0.39	-0.40	0.51	0.39
	Peak Flexion	0.73	0.88	0.26	0.26	0.84	0.75

TABLE S-VII
Stand-To-Sit Symmetry

		Subject 1		Subject 2		Subject 3	
		Powered	Passive	Powered	Passive	Powered	Passive
GRF	Vertical	0.29	0.31	0.27	0.35	0.31	0.24
	Propulsion	0.38	0.75	0.01	0.62	0.79	0.14
	Braking	-0.60	-0.05	-0.44	0.13	-0.07	0.29
Moment	Peak Abduction	0.83	0.75	-0.80	0.47	0.63	-0.16
	Peak Adduction	-0.06	-0.15	0.82	0.75	0.64	0.04
	Peak Extension	0.75	0.45	0.90	0.22	0.67	0.78
	Peak Flexion	-0.01	0.03	-0.30	0.03	-0.31	-0.18
Power	Peak Abduction	0.29	0.02	0.83	0.72	0.79	0.34
	Peak Adduction	0.53	-0.24	-0.10	0.70	0.59	0.07
	Peak Extension	0.84	0.66	0.57	0.40	0.91	0.66
	Peak Flexion	0.02	0.05	-0.41	-0.45	0.06	0.11

TABLE S-VIII
LEVEL WALKING SYMMETRY

		Subject 1		Subject 2		Subject 3	
		Powered	Passive	Powered	Passive	Powered	Passive
GRF	Vertical	0.09	0.02	0.08	0.02	0.13	0.05
	Propulsion	0.16	0.35	0.22	0.31	0.14	0.27
	Braking	0.22	0.08	0.31	0.20	-0.31	0.00
Moment	Peak Abduction	0.53	0.36	0.18	0.67	0.67	0.46
	Peak Adduction	0.11	0.06	0.18	0.13	0.38	0.22
	Peak Extension	0.20	0.61	-0.18	0.20	0.18	0.54
	Peak Flexion	-0.13	-0.24	0.11	-0.21	0.11	-0.16
Power	Peak Abduction	0.14	0.11	0.11	0.06	0.53	0.37
	Peak Adduction	0.46	0.11	0.35	-0.08	0.35	0.45
	Peak Extension	-0.20	-0.12	-0.33	-0.29	0.01	-0.06
	Peak Flexion	0.06	-0.09	-0.29	0.07	0.42	0.08

TABLE S-IX
STAIR DESCENT SYMMETRY

		Subject 1		Subject 2		Subject 3	
		Powered	Passive	Powered	Passive	Powered	Passive
GRF	Vertical	0.28	0.18	0.14	0.05	0.07	0.18
	Propulsion	0.22	-0.20	0.34	0.92	-0.18	-0.22
	Braking	0.45	0.57	-0.40	0.02	0.72	0.61
Moment	Peak Abduction	-0.11	0.02	0.67	0.86	0.02	-0.03
	Peak Adduction	0.63	0.53	0.20	0.03	0.58	0.56
	Peak Extension	0.26	0.08	-0.08	0.15	-0.12	0.48
	Peak Flexion	-0.28	-0.53	0.33	0.46	-0.01	-0.20
Power	Peak Abduction	0.62	0.50	0.47	0.15	0.70	0.34
	Peak Adduction	0.45	0.71	0.32	0.74	0.57	0.34
	Peak Extension	-0.27	-0.07	0.40	0.84	-0.67	-0.04
	Peak Flexion	-0.14	-0.28	0.32	0.63	0.07	-0.31

TABLE S-X
STAIR ASCENT SYMMETRY

		Subject 1		Subject 2		Subject 3	
		Powered	Passive	Powered	Passive	Powered	Passive
GRF	Vertical	0.05	0.18	0.11	-0.11	0.06	0.16
	Propulsion	0.02	0.45	0.40	-0.21	0.65	0.75
	Braking	0.42	0.76	0.23	0.99	-0.65	-0.20
Moment	Peak Abduction	0.12	-0.25	0.62	0.22	0.61	-0.13
	Peak Adduction	0.01	0.21	0.38	-0.20	0.59	0.01
	Peak Extension	0.41	0.43	0.34	0.40	0.58	0.39
	Peak Flexion	-0.03	0.00	-0.24	-0.16	-0.28	-0.11
Power	Peak Abduction	0.04	0.05	0.55	-0.33	0.85	0.18
	Peak Adduction	-0.48	0.54	0.30	0.59	-0.04	-0.19
	Peak Extension	-0.53	0.23	-0.16	0.39	-0.13	0.31
	Peak Flexion	0.20	0.27	0.20	0.54	0.61	0.15

References

[1] D. A. Winter, *Biomechanics and motor control of human movement*, 4th ed. Hoboken, N.J: Wiley, 2009.

[2] E. Reznick, K. R. Embry, R. Neuman, E. Bolívar-Nieto, N. P. Fey, and R. D. Gregg, "Lower-limb kinematics and kinetics during continuously varying human locomotion," *Scientific Data*, vol. 8, no. 1, p. 282, 2021.

[3] J. Camargo, A. Ramanathan, W. Flanagan, and A. Young, "A comprehensive, open-source dataset of lower limb biomechanics in multiple conditions of stairs, ramps, and level-ground ambulation and transitions," *J. Biomechanics*, vol. 119, p. 110320, 2021.

[4] K. Scherpereel, D. Molinaro, O. Inan, M. Shepherd, and A. Young, "A human lower-limb biomechanics and wearable sensors dataset during cyclic and non-cyclic activities," *Scientific Data*, vol. 10, no. 1, p. 924, 2023.

[5] S. Viteckova, P. Kutilek, Z. Svoboda, R. Krupicka, J. Kauler, and Z. Szabo, "Gait symmetry measures: A review of current and prospective methods," *Biomedical Signal Processing and Control*, vol. 42, pp. 89–100, 2018.
